# Supplementary material for: Optimization of experimental designs for biological rhythm discovery
Source: PLoS Comput Biol. 2025 Nov 10;21(11):e1013662. doi: 10.1371/journal.pcbi.1013662 (PMC12617917; doi:10.1371/journal.pcbi.1013662)
Supplement: S1 Text — (PDF) [file pcbi.1013662.s001.pdf]

S1-1 Mathematical proofs of results

S1-1.1 Closed-form power expression

The power formula in Theorem 3.1 follows from computing the distribution of the F-statistic under the null and alternative models. We show these computations in detail since self-contained proofs are unavailable in the literature. The first three lemmas are concerned with elementary properties of the design matrix X and the hypothesis matrix H.

Lemma S1-1.1. *Let $X = X(\mathbf{t}; f) \in \mathbb{R}^{N\times 3}$ be the design matrix of the cosinor model and suppose at least three components of the vector $\mathbf{z} = e^{2\pi i f \mathbf{t}} \in \mathbb{C}^N$ are distinct, then X has full column rank.*

Proof. Without loss of generality, assume the first three components z_1, z_2, z_3 of $\mathbf{z} \in \mathbb{C}^N$ are distinct. Let $\theta_1, \theta_2, \theta_3 \in [0, 2\pi)$ be the corresponding angles $\theta_j = \mathrm{Arg}(z_j)$. To verify that X is of full column rank, it suffices to show that the submatrix

$$M = \begin{bmatrix} 1 & \cos(\theta_1) & \sin(\theta_1) \\ 1 & \cos(\theta_2) & \sin(\theta_2) \\ 1 & \cos(\theta_3) & \sin(\theta_3) \end{bmatrix} \tag{S1}$$

has nonzero determinant. Assume without loss of generality that $\theta_3 = 0$ and compute the determinant of M to obtain

$$\det M = \sin(\theta_1) - \sin(\theta_2) - \sin(\theta_1)\cos(\theta_2) + \cos(\theta_1)\sin(\theta_2). \tag{S2}$$

Suppose towards contradiction that $\det M = 0$ and rearrange Eq S2 to show

$$\frac{\sin(\theta_1)}{1-\cos(\theta_1)} = \frac{\sin(\theta_2)}{1-\cos(\theta_2)}. \tag{S3}$$

The restriction of the function $f(x) = \frac{\sin(x)}{1-\cos(x)}$ to $x \in [0, 2\pi)$ is strictly monotone and therefore injective. Hence Eq S3 implies $\theta_1 = \theta_2$, a contradiction. □

Lemma S1-1.2. *Let $X \in \mathbb{R}^{n\times p}$ with $\mathrm{rank}(X) = p \leq n$ then $X^T X$ is symmetric positive definite.*

Proof. Since $X^T X$ is symmetric, there exists an orthonormal eigenbasis $\mathbf{v}_1, \ldots, \mathbf{v}_p$ and real eigenvalues $\xi_1, \ldots, \xi_p$

such that $X^T X\mathbf{v}_i = \xi_i \mathbf{v}_i$ for $i = 1, \ldots, p$. Since $\mathrm{rank}(X) = p$, we know $X\mathbf{v}_i \neq 0$ and so

$$0 < ||X\mathbf{v}_i||_2^2 = \mathbf{v}_i^T X^T X \mathbf{v}_i = \xi_i ||\mathbf{v}_i||_2^2, \tag{S4}$$

which forces $\xi_i > 0$ for $i = 1, \ldots, p$. $\square$

Lemma S1-1.3. *Suppose $A \in \mathbb{R}^{p\times p}$ is symmetric positive definite and $H \in \mathbb{R}^{q\times p}$ with $\mathrm{rank}(H) = q$. If $q \leq p$ then $B = HAH^T$ is symmetric positive definite.*

Proof. Symmetry is apparent, so it suffices to show that $\mathbf{x}^T HAH^T\mathbf{x} > 0$ for any $\mathbf{x} \in \mathbb{R}^q$. Since $\mathrm{rank}(H) = q$, we know $H^T\mathbf{x} \neq 0$ for all nonzero $\mathbf{x} \in \mathbb{R}^q$. Positivity of HAH^T now follows from the positivity of A, since $(\mathbf{x}^T H)A(H^T\mathbf{x}) > 0$ for all $\mathbf{x} \in \mathbb{R}^q$. $\square$

The next lemma and its corollary will be useful for computing the null and alternative distributions of the F statistic. In particular, it is useful to recognise that the definition of the F-statistic most frequently applied in experiments can also be written in a form more suitable for mathematical proofs.

Lemma S1-1.4. *Given a design matrix $X \in \mathbb{R}^{n\times p}$ with $\mathrm{rank}(X) = p$ and a hypothesis matrix $H \in \mathbb{R}^{q\times p}$ with $q \leq p \leq n$, define the following linear subspaces*

- $V_u = \{X\boldsymbol{\beta} : \boldsymbol{\beta} \in \mathbb{R}^p\} = \mathrm{Col}(X)$,
- $V_r = \{X\boldsymbol{\beta} : \boldsymbol{\beta} \in \mathbb{R}^p, H\boldsymbol{\beta} = 0\}$,
- $V_\ell = V_u \cap V_r^\perp$, *the orthogonal complement of V_r within V_u.*

Let P_u, P_r, and P_ℓ be the orthogonal projectors onto the respective subspaces. The projector P_ℓ satisfies

$$P_\ell = P_u - P_r = Q(Q^T Q)^{-1} Q^T \tag{S5}$$

in which $Q = X(X^T X)^{-1} H^T$.

Proof. Since V_u can be expressed as $V_u = V_\ell \oplus V_r$, we clearly have $P_\ell = P_u - P_r$. To verify the second equality, we must show that the matrix $M = Q(Q^T Q)^{-1} Q^T$ is equal to the orthogonal projector corresponding to V_ℓ. The properties $M^2 = M$ and $M^T = M$ which verify that M is an orthogonal projector follow immediately from the definition of Q. We verify using elementary facts from linear algebra that the action of M and P_ℓ coincide for all $\mathbf{v} \in V_\ell$. Notice that we may write $\mathbf{v} = X\boldsymbol{\gamma}$ for some $\boldsymbol{\gamma} \in \mathbb{R}^p$, and given any $\boldsymbol{\beta} \in \mathbb{R}^p$ with $H\boldsymbol{\beta} = 0$, we must have

$$\mathbf{v}^T X\boldsymbol{\beta} = 0 = \boldsymbol{\gamma}^T X^T X\boldsymbol{\beta}. \tag{S6}$$

Eq S6 implies $X^T X\boldsymbol{\gamma} \in \text{Col}(H^T)$, and so$X^T X\boldsymbol{\gamma} = H^T\boldsymbol{\alpha}$ for some $\boldsymbol{\alpha} \in \mathbb{R}^q$. It follows that $\mathbf{v} = X\boldsymbol{\gamma} = Q\boldsymbol{\alpha}$ and thus

$$M\mathbf{v} = Q(Q^TQ)^{-1}Q^TQ\boldsymbol{\alpha} = Q\boldsymbol{\alpha} = \mathbf{v}. \tag{S7}$$

Similar reasoning shows that M agrees with P_ℓ on $V_\ell^\perp$, completing the proof. Indeed if $\mathbf{w} \in V_\ell^\perp$ then $\mathbf{w}$ can be written as

$$\mathbf{w} = \mathbf{u} + X\boldsymbol{\rho}, \tag{S8}$$

with $\mathbf{u} \in V_u^\perp$, $X\boldsymbol{\beta} \in V_r$, and $H\boldsymbol{\rho} = 0$. We have that $Q^T\mathbf{u} = 0$ since

$$\mathbf{u} \in \text{Col}(X)^\perp = \text{Null}(X^T). \tag{S9}$$

Similarly we find $Q^T\mathbf{r} = H\boldsymbol{\rho} = 0$. □

Corollary S1-1.4.1. *Let $X = X(\mathbf{t}; f)$ be the design matrix of the cosinor model. Given data $\mathbf{y} \in \mathbb{R}^n$, the total sum of squares*

$$\text{TSS} = ||\mathbf{y} - \langle\mathbf{y}\rangle||^2 = ||(I - P_r)\mathbf{y}||^2, \tag{S10}$$

and the residual sum of squares

$$\text{RSS} = ||\mathbf{y} - X\hat{\boldsymbol{\beta}}||^2 = ||(I - P_u)\mathbf{y}||^2, \tag{S11}$$

in which $\hat{\boldsymbol{\beta}} = (X^TX)^{-1}X^T\mathbf{y}$ is the least squares estimator and X is the one-frequency cosinor design matrix, the following three definitions of the F-statistic are equivalent

$$\hat{F}_1 = \frac{TSS - RSS}{RSS}\frac{N-p}{p-1}, \tag{S12}$$

$$\hat{F}_2 = \frac{\mathbf{y}^T\left(P_u - P_r\right)\mathbf{y}}{\mathbf{y}^T\left(I - P_u\right)\mathbf{y}}\frac{N-p}{p-1}, \tag{S13}$$

$$\hat{F}_3 = \frac{\left(H\hat{\boldsymbol{\beta}}\right)^T\left(H\left(X^TX\right)^{-1}H^T\right)^{-1}\left(H\hat{\boldsymbol{\beta}}\right)}{\mathbf{y}^T\left(I - P_u\right)\mathbf{y}}\frac{N-p}{p-1}, \tag{S14}$$

in which $H = \begin{bmatrix} 0 & 1 & 0 \\ 0 & 0 & 1 \end{bmatrix}$ is the rhythm-detection hypothesis matrix.

The linear algebraic tools assembled so far allow us to deduce the null and alternative distributions of the F-statistic by simply applying Cochran's theorem.

Theorem S1-1.5 (Cochran (1934), [1])**.** *If* $\mathbf{x} \in \mathbb{R}^n \sim \mathcal{N}(\mathbf{0}, I)$ *and a family of* $n \times n$ *matrices* $\{A_i\}_{i=1}^m$ *satisfy*

1. $A_i = A_i^T$ *for* $i = 1, \dots, m$,
2. $\operatorname{rank}(A_i) = r_i$ *for* $i = 1, \dots, m$,
3. $\sum_{i=1}^m A_i = I$,

then each $\mathbf{x}^T A_i \mathbf{x} \sim \chi^2_{r_i}$ *independently if and only if* $\sum_{i=1}^m r_i = n$.

Proof. See Chapter 2 of [2]. □

Lemma S1-1.6 (Null distribution of F-statistic)**.** *Suppose data* $\mathbf{y} \in \mathbb{R}^n$ *is of the form* $\mathbf{y} = X\boldsymbol{\beta} + \boldsymbol{\varepsilon} \in \mathbb{R}^N$ *with* $\boldsymbol{\varepsilon} \sim \mathcal{N}(\mathbf{0}, \sigma^2 I)$ *and the null hypothesis* $H\boldsymbol{\beta} = 0$ *holds true, then the F-statistic from Corollary S1-1.4.1 satisfies*

$$\hat{F}(\mathbf{y}) \sim F(\cdot; 2, N-3). \tag{S15}$$

Proof. We use the definition of the F-statistic in Eq S13. Since the projection matrices $(I - P_u)$ and $(P_u - P_r)$ vanish on constant vectors, we may assume without loss of generality that $\boldsymbol{\mu} = \mathbf{0}$. Since the projection matrices are idempotent and symmetric, their ranks are given by the dimension of the subspaces onto which they project

$$\operatorname{rank}(P_u - P_r) = p - 1 = 2, \quad \operatorname{rank}(I - P_u) = N - p = N - 3. \tag{S16}$$

It follows from Theorem S1-1.5 that the following two quadratic forms have independent chi-squared distributions

$$\mathbf{y}^T (P_u - P_r) \mathbf{y} \sim \chi^2(2) \tag{S17}$$

$$\mathbf{y}^T (I - P_u) \mathbf{y} \sim \chi^2(N-3), \tag{S18}$$

and hence their ratio has an $F(\cdot; 2, N-3)$ distribution after scaling by the degrees of freedom. □

Lemma S1-1.7. *Given commuting projection matrices* P_A *and* P_B *that satisfy* $P_A P_B = 0$ *and* $\mathbf{x} \sim \mathcal{N}(\boldsymbol{\mu}, \sigma^2 I)$, *the random variables* $||P_A \mathbf{x}||_2^2$ *and* $||P_B \mathbf{x}||_2^2$ *are independent.*

Proof. Since P_A and P_B are commuting projectors they can be simultaneously diagonalized. Working in their

common eigenbasis, their characteristic function $\chi_{AB}(||P_A\mathbf{x}||_2^2, ||P_B\mathbf{x}||_2^2)$ clearly satisfies

$$\chi(||P_A\mathbf{x}||_2^2, ||P_B\mathbf{x}||_2^2) = \chi_A(||P_A\mathbf{x}||_2^2)\chi_B(||P_B\mathbf{x}||_2^2) \tag{S19}$$

which implies independence. □

Lemma S1-1.8 (Alternative distribution of F-statistic)**.** *Suppose data $\mathbf{y} \in \mathbb{R}^N$ is of the form $\mathbf{y} = X\boldsymbol{\beta} + \boldsymbol{\varepsilon} \in \mathbb{R}^N$ with $\boldsymbol{\varepsilon} \sim \mathcal{N}(\mathbf{0}, \sigma^2 I)$ and the alternative hypothesis $H\boldsymbol{\beta} \neq 0$ holds true, then the F-statistic from Corollary S1-1.4.1 has a noncentral F-distribution*

$$\hat{F}(\mathbf{y}) \sim F_\lambda(\cdot|2, N-3) \tag{S20}$$

with noncentrality parameter $\lambda = \frac{1}{\sigma^2}\left(H\boldsymbol{\beta}\right)^T B^{-1} H\boldsymbol{\beta}$ in which $B = H(X^TX)^{-1}H^T$.

Proof. The vector appearing in the numerator of the F-statistic in Eq S13 satisfies

$$H\hat{\boldsymbol{\beta}} \sim \mathcal{N}(H\boldsymbol{\beta}, \sigma^2 H(X^TX)^{-1}H^T). \tag{S21}$$

Since X and H satisfy the non-degeneracy conditions of Lemmas S1-1.1-S1-1.3, B is symmetric positive definite and therefore has a Cholesky factorization. Let $B^{-1/2}$ be a Cholesky factor of B^{-1}, and since $\frac{1}{\sigma}B^{-1/2}H\hat{\boldsymbol{\beta}} \sim \mathcal{N}(B^{-1/2}H\boldsymbol{\beta}, I)$, the numerator of Eq S13 can be rescaled to have a noncentral chi-squared distribution

$$\frac{1}{\sigma^2}(H\hat{\boldsymbol{\beta}})^T B^{-1}(H\hat{\boldsymbol{\beta}}) \sim \chi^2_\lambda(2) \tag{S22}$$

with noncentrality parameter $\lambda = \frac{1}{\sigma^2}(H\boldsymbol{\beta})^T B^{-1} H\boldsymbol{\beta}$. Since $(I - P_u)X\boldsymbol{\beta} = 0$ for any $\boldsymbol{\beta} \in \mathbb{R}^p$, the denominator of Eq S13 satisfies

$$\frac{1}{\sigma^2}\left(\mathbf{y} - X\boldsymbol{\beta}\right)^T\left(I - P_u\right)\left(\mathbf{y} - X\boldsymbol{\beta}\right) = \boldsymbol{\varepsilon}^T(I - P_u)\boldsymbol{\varepsilon} \sim \chi^2(N-3). \tag{S23}$$

Finally Lemma S1-1.7 ensures that the numerator and denominator of Eq S13 are independent and the claim follows. □

Proof of Theorem 3.1. The power γ of a hypothesis test is given by

$$\gamma(\boldsymbol{\beta}) = \mathbb{P}_{\boldsymbol{\beta}}(\hat{F}(\mathbf{y}) \in R) \tag{S24}$$

in which $R \subset \mathbb{R}^N$ is the rejection region, $\mathbf{y} \in \mathbb{R}^N$ is the data and $\hat{F}$ is the F-statistic. Lemma S1-1.6 implies $R = \{\mathbf{y} \in \mathbb{R}^N : \hat{F}(\mathbf{y}) \geq F^{-1}(1-\alpha|2, N-3)\}$ and we may conclude from Lemma S1-1.8 that $\mathbb{P}_{\boldsymbol{\beta}}(x \geq c) = 1 - F_\lambda(c; 2, N-3)$ for any $c > 0$. Hence we have

$$\gamma(\boldsymbol{\beta}) = 1 - F_\lambda(F_0^{-1}(1-\alpha; 2, N-3); 2, N-3) \tag{S25}$$

with $\lambda = \frac{1}{\sigma^2}(H\boldsymbol{\beta})^T B^{-1} H\boldsymbol{\beta}$. □

S1-1.2 Monotonicity of the noncentral F-distribution

Throughout our work, we treat maximization of power and maximization of the noncentrality parameter as equivalent optimization problems. This equivalence is justified because the F-distribution is a monotone function of its noncentrality parameter.

Proposition S1-1.1 (Monotonicity of the noncentral F distribution)**.** *The noncentral F distribution $F_\lambda(x; n_1, n_2)$ with degrees of freedom $n_1 = 2$ and $n_2 = N - 3 > 0$ is a monotone decreasing function of the noncentrality parameter λ.*

We prove Prop. S1-1.1 by expressing the F-distribution as an infinite series, justifying term-wise differentiation, and verifying that the derivative of the distribution with respect to its noncentrality parameter has a definite sign.

Lemma S1-1.9 (Series representation of noncentral F-distribution)**.** *The cumulative distribution function $F(x; n_1, n_2, \lambda)$ for a noncentral F-distributed random variable with degrees of freedom n_1, n_2 and noncentrality parameter λ can be expressed as a series*

$$F(x; n_1, n_2, \lambda) = \sum_{k=0}^{\infty} \left(\frac{\left(\frac{\lambda}{2}\right)^k}{k!} e^{-\lambda/2} \right) I\left(\frac{n_1 x}{n_2 + n_1 x}; \frac{n_1}{2} + k, \frac{n_2}{2} \right), \tag{S26}$$

in which $I(z; a, b)$ is the regularized incomplete beta function, defined by

$$B(z; a, b) := \int_0^z t^{a-1}(1-t)^{b-1} \, \mathrm{d}t, \tag{S27}$$

$$I(z; a, b) := B(z; a, b)/B(1; a, b). \tag{S28}$$

Proof. A derivation of this formula is given in [3]. □

Lemma S1-1.10. *For $z \in [0,1]$ and $a > 0, b > 0$, the regularized incomplete beta functions obey*

- $I(z; a+1, b) \leq I(z, a, b)$,
- $\lim_{a\to\infty} I(z; a+1, b)/I(z; a, b) = z$.

Proof. The coefficient $B(1, a, b)$ in Eq S28 can be expressed as

$$B(1,a,b) = \frac{\Gamma(a)\Gamma(b)}{\Gamma(a+b)} \tag{S29}$$

in which $\Gamma(z) = \int_0^\infty t^{z-1}e^{-t}\mathrm{d}t$ is the gamma function [4]. Use integration by parts and the identity $\Gamma(z+1) = z\Gamma(z)$ to obtain

$$I(z; a+1, b) = I(z; a, b) - \frac{z^a(1-z)^b}{aB(1; a, b)}. \tag{S30}$$

Since $z \in [0,1]$ the final term in Eq S30 is of definite sign, hence

$$I(z; a+1, b) \leq I(z; a, b). \tag{S31}$$

To obtain the asymptotic result, rearrange Eq S30 and integrate by parts to obtain

$$\begin{aligned}
\frac{I(z; a+1, b)}{I(z; a, b)} &= 1 - \frac{z^a(1-z)^b}{a\int_0^z x^{a-1}(1-x)^{b-1}\mathrm{d}x} && \text{(S32)}\\
&= 1 - \frac{z^a(1-z)^b}{z^a(1-z)^{b-1} + (b-1)\int_0^z x^a(1-x)^{b-2}\mathrm{d}x} && \text{(S33)}\\
&= 1 - \frac{1-z}{1 + \frac{(b-1)}{(1-z)^{b-1}}\int_0^z \left(\frac{x}{z}\right)^a (1-x)^{b-2}\mathrm{d}x}. && \text{(S34)}
\end{aligned}$$

Since $b > 0$, $x < z$ and $z \in [0,1]$, we have

$$\int_0^z \left(\frac{x}{z}\right)^a (1-x)^{b-2}\mathrm{d}x \leq \int_0^z (1-x)^{b-2}\mathrm{d}z = \begin{cases} -\log(1-z) & b = 1 \\ \frac{1-(1-z)^{b-1}}{b-1} & \text{otherwise} \end{cases}, \tag{S35}$$

and hence we can evaluate the desired limit using the dominated convergence theorem to obtain

$$\lim_{a\to\infty} \int_0^z \left(\frac{x}{z}\right)^a (1-x)^{b-2}\mathrm{d}x = 0. \tag{S36}$$

So we conclude from Eq S34 that $\lim_{a\to\infty} I(z;a+1,b)/I(z;a,b) = z$. $\square$

Lemma S1-1.11. *Let $T_k(z, n_1, n_2, \lambda)$ be the terms of the series expansion in Eq S26 in which $z = \frac{n_1 x}{n_1 x + n_2}$ and $\lambda > 0$. The terms $T_k(z, n_1, n_2, \lambda)$ satisfy the following:*

- $\sum_{k=0}^{\infty} T_k(z; n_1, n_2, \lambda)$ *converges,*
- $\sum_{k=0}^{\infty} \partial_\lambda T_k(z, n_1, n_2, \lambda)$ *converges uniformly for all $\lambda > 0$.*

Proof. Convergence of $\sum_{k=0}^{\infty} T_k(z, n_1, n_2, \lambda)$ follows from the ratio test

$$\frac{T_{k+1}(z, n_1, n_2, \lambda)}{T_k(z, n_1, n_2, \lambda)} = \frac{I_{k+1}}{I_k} \frac{\lambda}{2(k+1)} \leq \frac{\lambda}{2(k+1)}, \tag{S37}$$

which implies $\lim_{k\to\infty} \left| \frac{T_{k+1}(z,n_1,n_2,\lambda)}{T_k(z,n_1,n_2,\lambda)} \right| = 0$ for any $\lambda > 0$. Uniform convergence of the derivatives can be verified using the Weierstrass M-test. To construct a majorant for $T_k(z, n_1, n_2, \lambda)$, notice that $\partial_\lambda T_k$ has critical points at $\lambda = 2k$ and $\lambda = 0$. We know that $T_k(z, n_1, n_2, 0) = 0$ and $\lim_{\lambda\to\infty} T_k(z, n_1, n_2, \lambda) = 0$ so

$$g_k(z, n_1, n_2) := T_k(z, n_1, n_2, 2k) = I_k \frac{k^k}{k!} e^{-k} \tag{S38}$$

must be the global maximum of $T_k(z, n_1, n_2, \lambda)$ on the interval $\lambda \in [0, \infty)$. This majorant also leads to a bound on the derivative, since for $k = 0$, we have $|\partial_\lambda T_0| \leq \frac{1}{2} T_0$ and for $k > 0$

$$|\partial_\lambda T_k| = \left| -\frac{1}{2} T_k + e^{-\lambda/2} \frac{\lambda^{k-1}}{(k-1)!2^k} I\left(z; \frac{n_1}{2} + k, \frac{n_2}{2}\right) \right| \tag{S39}$$

$$\leq \frac{1}{2} T_k + \frac{1}{2} e^{-\lambda/2} \frac{\lambda^{k-1}}{(k-1)!2^{k-1}} I\left(z; \frac{n_1}{2} + k, \frac{n_2}{2}\right) \tag{S40}$$

$$\leq \frac{1}{2} T_k + \frac{1}{2} e^{-\lambda/2} \frac{\lambda^{k-1}}{(k-1)!2^{k-1}} I\left(z; \frac{n_1}{2} + k - 1, \frac{n_2}{2}\right) \tag{S41}$$

$$= \frac{1}{2} \left(T_k + T_{k-1} \right). \tag{S42}$$

To prove uniform convergence of $\sum_k \partial_\lambda T_k$, it remains to show that $\lim_{k\to\infty} \left| \frac{g_{k+1}}{g_k} \right| < 1$. Indeed, we have

$$\frac{g_{k+1}}{g_k} = \frac{I_{k+1}}{I_k} \frac{(k+1)^{k+1}}{k^k} \frac{k!}{(k+1)!} e^{-1} = \frac{I_{k+1}}{I_k} \left(\frac{k+1}{k} \right)^k e^{-1} = \frac{I_{k+1}}{I_k} e^{-1} \left(1 + \frac{1}{k} \right)^k. \tag{S43}$$

We know $\lim_{k\to\infty} \left(1 + \frac{1}{k}\right)^k = e$ and so

$$\lim_{k\to\infty} \left| \frac{g_{k+1}}{g_k} \right| = \lim_{k\to\infty} \frac{I_{k+1}}{I_k} = z < 1, \tag{S44}$$

with the last equality justified by the second claim of Prop. S1-1.10. □

Proof of Proposition S1-1.1. Use Prop. S1-1.9 to express $F(x; n_1, n_2, \lambda)$ as

$$F(x; n_1, n_2, \lambda) = \sum_{k=0}^{\infty} T_k(z, n_1, n_2, \lambda) \tag{S45}$$

in which $z = \frac{n_1 x}{n_1 x + n_2}$. Since $n_2 = N - 3 > 0$ by assumption, it follows that $z \in [0, 1]$ and we may apply Lemma S1-1.10 and Lemma S1-1.11 to justify term-wise differentiation of the series. Differentiate term-wise and apply the same reasoning as in the proof of Lemma S1-1.11 to find

$$\frac{\partial}{\partial \lambda} F(x; n_1, n_2, \lambda) \leq -\frac{1}{2} T_0(z; n_1, n_2, \lambda) + \sum_{k=1}^{\infty} \frac{1}{2} \left(-T_k(z; n_1, n_2, \lambda) + T_{k-1}(z; n_1, n_2, \lambda) \right) \tag{S46}$$

$$= \lim_{N \to \infty} -\frac{1}{2} T_N(z; n_1, n_2, \lambda) \leq 0, \tag{S47}$$

which verifies that $F(x; n_1, n_2, \lambda)$ is monotone decreasing for all $\lambda \geq 0$. □

S1-1.3 Worst-case power maximization

We prove Theorem 3.2 in two steps. We first show that the worst-case value of the noncentrality parameter is related to an eigenvalue of the matrix $B(\mathbf{t}; f)$ defined in Eq 13. We then compute this eigenvalue for equispaced designs and show that it is optimal using a classical result from optimal experimental design.

Lemma S1-1.12. *Let H be the hypothesis matrix defined in Eq 11 and let $B = B(\mathbf{t}; f)$ be the matrix given in Eq 13. The lowest value of the noncentrality parameter across all signals of a given amplitude and frequency*

$$\lambda^* := \min_{\beta_1^2 + \beta_2^2 = A^2} \frac{1}{\sigma^2} \boldsymbol{\beta}^T H^T B^{-1} H \boldsymbol{\beta} \tag{S48}$$

is given by

$$\lambda^* = \frac{A^2}{\sigma^2} \xi_{\min}(B^{-1}), \tag{S49}$$

in which $\xi_{\min}(\cdot)$ is the smallest eigenvalue of a symmetric matrix.

Proof. Start by simplifying the definition of λ^* as

$$\lambda^* = \min_{\beta_1^2+\beta_2^2=A^2} \frac{1}{\sigma^2}\boldsymbol{\beta}^T H^T B^{-1} H\boldsymbol{\beta} = \min_{\beta_1^2+\beta_2^2=A^2} \frac{1}{\sigma^2} \begin{bmatrix}\beta_1 & \beta_2\end{bmatrix} B^{-1} \begin{bmatrix}\beta_1 \\ \beta_2\end{bmatrix} \tag{S50}$$

$$= \frac{A^2}{\sigma^2} \min_{\beta_1^2+\beta_2^2=1} \begin{bmatrix}\beta_1 & \beta_2\end{bmatrix} B^{-1} \begin{bmatrix}\beta_1 \\ \beta_2\end{bmatrix}. \tag{S51}$$

We can set $\frac{A}{\sigma} = 1$ without loss of generality since it appears homogeneously in Eq S51. Lemma S1-1.2 ensures that $(X^T X)^{-1}$ is symmetric positive definite when X is the design matrix for the cosinor model. Since the rhythm detection hypothesis matrix has full row rank, Lemma S1-1.3 ensures that B is symmetric positive definite. It follows that B^{-1} has a Cholesky factorization $B^{-1} = LL^T$ for some $L \in \mathbb{R}^{2\times 2}$. We may rewrite Eq S51 in terms of L and obtain

$$\lambda^* = \min_{\beta_1^2+\beta_2^2=1} \left|\left| L \begin{bmatrix}\beta_1 & \beta_2\end{bmatrix}^T \right|\right|^2 = \sigma_{\min}(L)^2. \tag{S52}$$

The last equality is justified by the max-min principle for singular values

$$\sigma_i(L) = \max_{\dim(U)=i} \min_{x\in U, ||x||=1} ||Lx||, \tag{S53}$$

with $\sigma_2 \leq \sigma_1$, so $i = 2$ gives the smallest singular value. The result follows by recalling that B^{-1} has real eigenvalues and thus $\sigma_{\min}(L)^2 = \xi_{\min}(B^{-1})$. □

The next two lemmas allow are used in computing the minimum eigenvalue of $B(\mathbf{t}; f)^{-1}$ for equispaced designs.

Lemma S1-1.13. *Let $B = B(\mathbf{t}; f)$ be the matrix $B = H(X^T X)^{-1} H^T$, in which $X = X(\mathbf{t}; f)$ is the design matrix for the one-frequency cosinor model and H is the hypothesis matrix. The inverse of B can be expressed as*

$$B^{-1} = \tilde{X}^T \tilde{X} - \frac{1}{N}\mathbf{b}\mathbf{b}^T, \tag{S54}$$

in which $\tilde{X} = \tilde{X}(\mathbf{t}; f) = \begin{bmatrix}\cos(2\pi f\mathbf{t}) & \sin(2\pi f\mathbf{t})\end{bmatrix}$ is the design matrix for the mean-free cosinor model $Y(t) = \beta_1 \cos(2\pi ft) + \beta_2 \sin(2\pi ft) + \varepsilon(t)$ and $\mathbf{b}$ is the vector of time-averages

$$\mathbf{b} = \mathbf{b}(\mathbf{t}; f) = \begin{bmatrix}\sum_{i=1}^N \cos(2\pi f t_i) & \sum_{i=1}^N \sin(2\pi f t_i)\end{bmatrix}^T. \tag{S55}$$

Proof. Compute the inverse of $X^T X$ using the Schur complement formula and act with H to find that the only

remaining term agrees with the right hand side of Eq S54. □

Lemma S1-1.14. *If $\mathbf{t} \in \mathbb{R}^N$ is equispaced over a cycle of frequency f and $N \geq 3$ then*

1. $\sum_{i=1}^{N} \cos(2\pi f t_j) = 0 = \sum_{j=1}^{N} \sin(2\pi f t_j)$,
2. $X(\mathbf{t}; f)^T X(\mathbf{t}; f) = \operatorname{diag}(N, N/2, N/2)$.

Proof. Since the measurements are equispaced over a cycle of frequency f, we may assume without loss of generality that they are located at the roots of unity $z_k = e^{2\pi i k/N}$ for $k = 0, \dots, N-1$. The time averages can be evaluated using a finite geometric series to find

$$\sum_{j=1}^{N} \cos(2\pi f t_j) + i \sin(2\pi f t_j) = \sum_{k=0}^{N-1} z_k = \sum_{k=0}^{N-1} \left(e^{2\pi i/N}\right)^k = \frac{1 - e^{2\pi i}}{1 - e^{2\pi i/N}} = 0. \tag{S56}$$

The same argument applied to the entries of the $X^T X$ reveals that $X^T X = \operatorname{diag}(N, N/2, N/2)$. □

It follows from Lemma S1-1.14 that any equispaced $\mathbf{t} \in \mathbb{R}^N$ with $N > 3$ will produce a design matrix that satisfies

$$\xi_{\min}(B(\mathbf{t}; f)^{-1}) = \xi_{\min}(\tilde{X}^T \tilde{X}) = \frac{N}{2}. \tag{S57}$$

We verify that the value $N/2$ on the right hand side of Eq S57 is globally optimal by using an equivalence theorem from optimal experimental design. These classical theorems are reviewed in [5]. For our purposes, it is convenient to use a more modern statement of the result, which we adapt from [6].

Theorem S1-1.15 (Elfving optimality condition, [6, Theorem 7.22])**.** *Let Ξ be the design space*

$$\Xi = \{(w_i, t_i)_{i=1}^d : d \in \mathbb{N}, \quad 0 \leq w_i \leq 1, \quad \sum_{i=1}^{N} w_i = 1, \quad t_i \in [0,1] \quad \text{for } i = 1, \dots, d\} \tag{S58}$$

and let $\mathcal{M}(\Xi)$ be the corresponding space of moment matrices

$$\mathcal{M}(\Xi) = \sum_{i=1}^{N} w_i \mathbf{f}(t_i) \mathbf{f}(t_i)^T \tag{S59}$$

in which $\mathbf{f}(t) = \begin{bmatrix} \cos(2\pi f t) & \sin(2\pi f t) \end{bmatrix}^T$ is the mean-free cosinor model. A moment matrix $M^* \in \mathcal{M}(\Xi)$ is

Elfving optimal in the sense that

$$\xi_{\min}(M^*) = \max_{M \in \mathcal{M}(\Xi)} \xi_{\min}(M) \tag{S60}$$

if and only if there exists a positive semidefinite matrix $E \in \mathbb{R}^{2\times 2}$ *such that* $\operatorname{tr}(E) = 1$ *and*

$$\mathbf{f}(t)^T E \mathbf{f}(t) \leq \xi_{\min}(M) \quad \textit{for all } t \in [0,1]. \tag{S61}$$

Proof of Theorem 3.1. Power and noncentrality parameter maximization for a given acrophase, frequency, and amplitude are equivalent by Prop. S1-1.1. Maximizing the noncentrality parameter across all acrophases is equivalent to maximizing the minimum eigenvalue of $B(\mathbf{t}; f)^{-1}$ by Lemma S1-1.12. It remains to show that equispaced designs with $N > 3$ measurements provide optimal solutions to the eigenvalue programming problem and that their power is acrophase-independent.

First use Lemma S1-1.13 to rewrite the eigenvalue problem as

$$\xi_{\min}(B^{-1}) = \inf_{||\mathbf{x}||=1} \mathbf{x}^T B^{-1} \mathbf{x} = \inf_{||\mathbf{x}||=1} \mathbf{x}^T \left(\tilde{X}^T \tilde{X} - \frac{1}{N} \mathbf{b}\mathbf{b}^T \right) \mathbf{x}. \tag{S62}$$

We clearly have $\mathbf{x}^T \left(\tilde{X}^T \tilde{X} - \frac{1}{N} \mathbf{b}\mathbf{b}^T \right) \mathbf{x} \leq \mathbf{x}^T \tilde{X}^T \tilde{X} \mathbf{x}$ for any $\mathbf{x} \in \mathbb{R}^2$, and consequently

$$\xi_{\min}(B^{-1}) = \mathbf{x}^T \left(\tilde{X}^T \tilde{X} - \frac{1}{N} \mathbf{b}\mathbf{b}^T \right) \mathbf{x} \leq \xi_{\min}(\tilde{X}^T \tilde{X}). \tag{S63}$$

If $\mathbf{t}$ is equispaced, then $\mathbf{b} = \mathbf{0}$ by Lemma S1-1.14 and so $B^{-1} = \tilde{X}^T \tilde{X}$ and we find

$$\xi_{\min}(B^{-1}) = \xi_{\min}(\tilde{X}^T \tilde{X}) = \frac{N}{2}. \tag{S64}$$

To apply Theorem S1-1.15 and complete the verification of global optimality, notice that every equispaced design $\mathbf{t}$ corresponds to a standardized design $\xi(w_i, t_i)_{i=1}^N \in \mathcal{M}(\Xi)$ with $w_i = 1/N$ with design matrix $M(\xi) = \operatorname{diag}(1/2, 1/2)$. Notice that we have

$$\mathbf{f}(t)^T M(\xi) \mathbf{f}(t) = f_1^2(t) + f_2^2(t) = 1 = \operatorname{tr}(M(\xi)) \tag{S65}$$

which verifies global optimality by Theorem S1-1.15. Finally, explicit computation confirms that the noncentrality

parameter of an equispaced design satisfies

$$\lambda(\mathbf{t}; \boldsymbol{\beta}, f, \sigma) = \frac{A^2 N}{2\sigma^2}, \tag{S66}$$

where $A^2 = \beta_1^2 + \beta_2^2$ is the amplitude. Notice that Eq S66 does not depend on the acrophase of the signal, as required. □

S1-1.4 Power analysis at the Nyquist rate

For a sample size N and equispaced design $\mathbf{t}_N$, power analysis at the Nyquist rate $f = \frac{N}{2}$ requires some additional consideration. Even if the sample size satisfies $N > 3$, the measurements will not cover sufficiently many distinct phases for the design matrix to be full rank. Hence, the methods developed so far in this section are not immediately applicable for computing the worst-case power of an equispaced design at the Nyquist rate. The following proposition allows us to circumvent this issue.

Proposition S1-1.2 (Noncentrality parameter at the Nyquist rate)**.** *For $N > 3$, the noncentrality parameter of an equispaced design $\mathbf{t}_N$ satisfies*

$$\lim_{f \to \frac{N}{2}} \xi_{\min}(B(\mathbf{t}_N; f)^{-1}) = 0 \tag{S67}$$

and hence the worst-case power is given by $\gamma = \alpha$, in which α is the type-I error rate.

Proof. Evaluate the entries of $B\left(\mathbf{t}_N; \frac{N}{2}\right)^{-1}$ using Eq S54 and a finite geometric series to obtain

$$B\left(\mathbf{t}_N; \frac{N}{2}\right)^{-1} = \begin{cases} \begin{bmatrix} N & 0 \\ 0 & 0 \end{bmatrix} & \text{if } N \text{ is even,} \\ \begin{bmatrix} N - \frac{1}{N} & 0 \\ 0 & 0 \end{bmatrix} & \text{if } N \text{ is odd.} \end{cases} \tag{S68}$$

Since eigenvalues are continuous with respect to entrywise matrix convergence (see for instance [7, Proposition 2.4.9.2]), it follows that

$$\lim_{f \to \frac{N}{2}} \xi_{\min}\left(B\left(\mathbf{t}_N; \frac{N}{2}\right)^{-1}\right) = 0. \tag{S69}$$

When the noncentrality parameter $\lambda = 0$, the power expression simplifies to

$$\gamma\left(\mathbf{t}_N; \boldsymbol{\beta}, \frac{N}{2}, \sigma\right) = 1 - F_0\left(F_0^{-1}(1-\alpha; 2, N-3); 2, N-3\right) = \alpha. \tag{S70}$$

□

S1-1.5 Unions of equispaced designs

Lemma S1-1.16 (Equispaced union)**.** *Consider a family of equispaced designs $\mathcal{T} = \{\mathbf{t}^{(1)}, \ldots, \mathbf{t}^{(K)}\}$ with each $\mathbf{t}^{(i)} \in [0,1]^{N_i}$. If the design matrix of each individual equispaced $\mathbf{t}^{(i)}$ satisfies the non-degeneracy condition from Lemma S1-1.1, then the union of the designs*

$$\mathbf{t} = \begin{bmatrix} \mathbf{t}^{(1)} \\ \vdots \\ \mathbf{t}^{(K)} \end{bmatrix} \tag{S71}$$

provides optimal phase independent power.

Proof. Use Lemma S1-1.13 to express the matrix $B(\mathbf{t})^{-1}$ as in Eq S54. The time-average vector $\mathbf{b}(\mathbf{t}; f)$ defined in Eq S55 vanishes since

$$\mathbf{b}(\mathbf{t}; f) = \begin{bmatrix} \sum_{i=1}^{K} \sum_{j=1}^{N_i} \cos(2\pi f t_j^{(i)}) \\ \sum_{i=1}^{K} \sum_{j=1}^{N_i} \sin(2\pi f t_j^{(i)}) \end{bmatrix} = \sum_{i=1}^{K} \mathbf{b}(\mathbf{t}^{(i)}; f) = \mathbf{0}. \tag{S72}$$

Hence, using Eq S54, matrix $B(\mathbf{t}, f) = H\left(X(\mathbf{t}; f)^T X(\mathbf{t}; f)\right)^{-1} H^T$ simplifies to

$$B(\mathbf{t}; f)^{-1} = \tilde{X}(\mathbf{t}; f)^T \tilde{X}(\mathbf{t}; f) = \mathrm{diag}(N/2, N/2) \tag{S73}$$

in which $N = \sum_{i=1}^{K} N_i$. It follows from Eq S73 that the phase-independent power and optimality properties of the individual designs are still applicable in the full design $\mathbf{t}$.

□

S1-1.6 Optimality for convex phase-invariant objectives

In this section we prove that the optimality of equispaced designs generalizes to a class of phase-invariant convex objective functions. We assume throughout this section that a single frequency f is under consideration. We use

the notation $\mathcal{P}(S_1)$ for the space of probability measures on the unit circle S_1 and use the notation $A \succ 0$ to denote that $A \in \mathbb{R}^{n \times n}$ is a symmetric positive definite matrix. The correspondence between designs and measures is made explicit in the definition below.

Definition S1-1.1 (Measure associated to experimental design)**.** For an experimental design $\mathbf{t} \in [0,1]^N$, the associated measure $\nu_{\mathbf{t}} \in \mathcal{P}(S_1)$ is given by

$$\nu_{\mathbf{t}}(x) = \frac{1}{N} \sum_{j=1}^{N} \delta(x - 2\pi f t_j), \tag{S74}$$

in which $\delta(\cdot)$ is the Dirac measure on S_1.

Since $\nu_{\mathbf{t}} \in \mathcal{P}(S_1)$ is a measure on the circle, it has natural notions of translation, translation invariance, and averaging with respect to the action of the circle group.

Definition S1-1.2 (Translation)**.** Given a measure $\mu \in \mathcal{P}(S_1)$ and $c \in [0, 2\pi)$, we use the notation μ_c to denote the translated measure $\mu_c(x) = \mu(x + c)$.

Definition S1-1.3 (Translation invariance)**.** A functional $f : \mathcal{P}(S_1) \to \mathbb{R}$ is translation invariant if it satisfies $f[\mu_c] = f[\mu]$ for all $\mu \in \mathcal{P}(S_1)$.

Definition S1-1.4 (Average measure)**.** Given $\mu \in \mathcal{P}(S_1)$ we define the average measure $\overline{\mu}$ by

$$\overline{\mu}(A) = \frac{1}{2\pi} \int_0^{2\pi} \mu(A + c) \ \mathrm{d}c \tag{S75}$$

for measurable $A \subset S_1$.

Lemma S1-1.17. *The average of Dirac measure coincides with the normalized Lebesgue measure on S_1.*

Proof. For Lebesgue measurable $A \subset S_1$, we have

$$\overline{\delta}(A) = \int_{S_1} \chi_A(x) \ \mathrm{d}\overline{\delta}(x) = \frac{1}{2\pi} \int_{S_1} \int_{S_1} \chi_A(x) \delta(x - c) \ \mathrm{d}c\mathrm{d}x \tag{S76}$$

$$= \frac{1}{2\pi} \int_{S_1} \left(\int_{S_1} \chi_A(x + c) \mathrm{d}c \right) \delta(x) \ \mathrm{d}x \tag{S77}$$

$$= \frac{|A|}{2\pi} \int_{S_1} \delta(x) \ \mathrm{d}x, \tag{S78}$$

$$= \frac{|A|}{2\pi} \tag{S79}$$

which coincides with the normalized Lebesgue measure of A. □

Since both measures in Lemma S1-1.17 are translation invariant, the result also follows from the uniqueness of normalized Haar measure on S_1 (see for instance Theorem 11.9 of [8]). The main result of this section, from which optimality of equispaced designs follows, is stated below.

Theorem S1-1.18. *Consider the functional $f : \mathcal{P}(S_1) \to \mathbb{R}$ given by*

$$f[\mu] = F\left(\int_{S_1} M(t; \boldsymbol{\beta})\ \mathrm{d}\mu(t)\right), \tag{S80}$$

in which $M : S_1 \times \mathbb{R}^p \to \mathbb{R}^{p\times p}$ is a matrix valued function with parameters $\boldsymbol{\beta} \in \mathbb{R}^p$ and $\mu \in \mathcal{P}(S_1)$. Suppose that f is translation invariant and $F : \mathbb{R}^{p\times p} \to \mathbb{R}$ is convex, then

$$f[\overline{\mu}] \leq f[\mu]. \tag{S81}$$

Proof. Apply Jensen's inequality to the averaged measure to obtain

$$\begin{aligned} f[\overline{\mu}] = F\left(\int_{S_1} M(t; \boldsymbol{\beta})\ \mathrm{d}\overline{\mu}(t)\right) &= F\left(\frac{1}{2\pi}\int_{S_1}\int_{S_1} M(t; \boldsymbol{\beta})\ \mathrm{d}\mu(t+c)\ \mathrm{d}c\right) && \text{(S82)} \\ &\leq \frac{1}{2\pi}\int_{S_1} F\left(\int_{S_1} M(t; \boldsymbol{\beta})\ \mathrm{d}\mu(t+c)\right) \mathrm{d}c && \text{(S83)} \\ &= \frac{1}{2\pi}\int_{S_1} f[\mu_c]\ \mathrm{d}c = \frac{1}{2\pi}\int_{S_1} f[\mu]\ \mathrm{d}c = f[\mu], && \text{(S84)} \end{aligned}$$

with the second last equality justified by the translation invariance of f. □

In order to apply Theorem S1-1.18 to power optimization, we verify that the problem can be formulated in terms of a translation invariant objective functional with convex dependence on the Fisher information matrix.

Lemma S1-1.19 (Matrix inversion is operator convex)**.** *The mapping $A \to A^{-1}$ is operator convex when restricted to the set of symmetric positive definite matrices.*

Proof. For $t \in [0, 1]$ and $A, B \succ 0$, we may write A has a unique square root $A^{1/2}$ by the Cholesky decomposition. We have

$$(tA + (1-t)B) = A^{-1/2}(tI + (1-t)C)^{-1}A^{-1/2}, \tag{S85}$$

in which $C = A^{-1/2}BA^{-1/2}$. Since I and C commute, they have a common eigenbasis and we can verify that for

any $\mathbf{x} \in \mathbb{R}^n$ such that $Cx = \xi x$, we have

$$(tI + (1-t)C)^{-1}x = \frac{1}{t + \xi(1-t)}x \leq tIx + (1-t)\frac{1}{\xi}x, \tag{S86}$$

by convexity of the map $r \to r^{-1}$ on the positive real line. Since $tI + (1-t)C$ is symmetric, it has a complete eigenbasis and it follows from Eq S86 that

$$(tI + (1-t)C)^{-1} \preceq tI^{-1} + (1-t)C^{-1}, \tag{S87}$$

and by conjugating by $A^{-1/2}$ we obtain

$$(tA + (1-t)B)^{-1} \preceq tA^{-1} + (1-t)B^{-1} \tag{S88}$$

as desired. □

Lemma S1-1.20 (Convexity of power optimization)**.** *The eigenvalue formulation of worst-case power maximization*

$$\mathbf{t}^* \in \arg\max_{\mathbf{t} \in [0,1]^N} \xi_{\min}(B(\mathbf{t}; f)^{-1}) \tag{S89}$$

is equivalent to

$$\mathbf{t}^* \in \arg\min_{\mathbf{t} \in [\mathbf{0},\mathbf{1}]^{\mathbf{N}}} \max_{f} \xi_{\max}(B(\mathbf{t}; f)), \tag{S90}$$

where $B(\mathbf{t}; f) = HM(\mathbf{t}; f)^{-1}H^T \in \mathbb{R}^{p \times p}$ and H is as given in Eq 11. Moreover, Eq S90 has convex dependence on the Fisher information matrix

$$M(\mathbf{t}; f) = X(\mathbf{t}; f)^T X(\mathbf{t}; f) \tag{S91}$$

Proof. Lemma S1-1.3 ensures that $B(\mathbf{t}; f)$ is symmetric positive definite and therefore satisfies $\xi_{\min}(B(\mathbf{t}; f)^{-1}) = \frac{1}{\xi_{\max}(B(\mathbf{t}; f))}$ and the equivalence follows. It remains to show that the map $M \to \xi_{\max}(HM^{-1}H^T)$ is convex when restricted to the set of symmetric positive definite matrices. For $M, N \succ 0$

and $s \in [0,1]$, Lemma S1-1.19 ensures that

$$(sM + (1-s)N)^{-1} \preceq sM^{-1} + (1-s)N^{-1}, \tag{S92}$$

so for any $\mathbf{x} \in \mathbb{R}^{p-1}$,

$$\mathbf{x}^T H(sM + (1-s)N)^{-1} H^T \mathbf{x} \leq s\left(\mathbf{x}^T H M^{-1} H^T \mathbf{x}\right) + (1-s)\left(\mathbf{x}^T H N^{-1} H^T \mathbf{x}\right). \tag{S93}$$

Convexity of the desired function follows from taking the maximum of Eq S93 over the unit ball. □

Lemma S1-1.21 (Invariance of eigenvalue optimization)**.** *Let $M(\mathbf{t}; f)$ be the Fisher information matrix associated to the design $\mathbf{t}$, the function*

$$F(M(\mathbf{t}; f)) = \xi_{\max}(HM(\mathbf{t}; f)^{-1} H^T) \tag{S94}$$

is invariant to phase shifts of the form $\mathbf{t} \to \mathbf{t} + \delta \mathbf{1}$ for $\delta \in \mathbb{R}$.

Proof. Fix $\delta \in \mathbb{R}$ and let $\tilde{\mathbf{t}} = \mathbf{t} + \delta\mathbf{1}$. The cosinor design matrix $X(\tilde{\mathbf{t}}; f)$ is related to $X(\mathbf{t}; f)$ through a unitary transformation

$$X(\tilde{\mathbf{t}}; f) = \begin{bmatrix} \mathbf{1} & \cos(2\pi f \tilde{\mathbf{t}}) & \sin(2\pi f \tilde{\mathbf{t}} \end{bmatrix} = X(\mathbf{t}; f) U_\phi \tag{S95}$$

where U_ϕ is of the form

$$U_\phi = \operatorname{diag}(1, R_\phi) = \begin{bmatrix} 1 & 0 & 0 \\ 0 & \cos\phi & \sin\phi \\ 0 & -\sin\phi & \cos\phi \end{bmatrix} \tag{S96}$$

with $\phi = 2\pi f \delta$. It follows that

$$B(\tilde{\mathbf{t}}; f) = HU_{-\phi} M(\mathbf{t}; f)^{-1} U_{-\phi}^T H^T = R_{-\phi} H M(\mathbf{t}; f)^{-1} H^T R_{-\phi}^T = R_{-\phi} B(\mathbf{t}; f) R_{-\phi}^T, \tag{S97}$$

which implies $\xi_{\max}(B(\tilde{\mathbf{t}}; f)) = \xi_{\max}(B(\mathbf{t}; f))$. □

Lemma S1-1.22. *Let $A, B : S_1 \to \mathbb{C}$ be functions of the form*

$$A(x) = \sum_{k \in K} a_k e^{ikx}, B(x) = \sum_{k \in K} b_k e^{ikx}, \tag{S98}$$

where $K \subset \mathbb{N}$ is finite. If there exists a collection of points $(x_j)_{j=1}^N \subset S_1$ such that

$$\sum_{j=1}^N e^{i(k-\ell)x_j} = 0 \tag{S99}$$

for all distinct $k, \ell \in K$, then

$$\sum_{j=1}^N A(x_j)\overline{B(x_j)} = \sum_{k \in K} a_k \overline{b_k} = \int_{S_1} A(x)\overline{B(x)}\ \mathrm{d}x. \tag{S100}$$

Proof. Expand the first sum in Eq S100and use Eq S99 to show that all interactions between frequencies e^{ikx} and $e^{i\ell x}$ vanish for $k \neq \ell$

$$\sum_{j=1}^N A(x_j)\overline{B(x_j)} = \sum_{j=1}^N \sum_{k,\ell \in K} a_k \overline{b_\ell} e^{i(k-\ell)x_j} = \sum_{k \in K} a_k \overline{b_k}. \tag{S101}$$

The second equality in Eq S100 is immediate from Parseval's theorem. □

Lemma S1-1.23. *Let $\mathbf{t} \in [0,1]^N$ with $N \geq 3$ with corresponding measure $\nu_{\mathbf{t}}$ from Eq S74. If $\mathbf{t}$ is equispaced, then the following two matrices coincide*

$$M(\nu_{\mathbf{t}}) = \int \begin{bmatrix} 1 & \cos(2\pi f t) & \sin(2\pi f t) \end{bmatrix} \begin{bmatrix} 1 \\ \cos(2\pi f t) \\ \sin(2\pi f t) \end{bmatrix} \mathrm{d}\nu_{\mathbf{t}}(t) = \mathrm{diag}\left(1, \frac{1}{2}, \frac{1}{2}\right), \tag{S102}$$

$$M_0 = \int \begin{bmatrix} 1 & \cos(2\pi f t) & \sin(2\pi f t) \end{bmatrix} \begin{bmatrix} 1 \\ \cos(2\pi f t) \\ \sin(2\pi f t) \end{bmatrix} \mathrm{d}t = \mathrm{diag}\left(1, \frac{1}{2}, \frac{1}{2}\right). \tag{S103}$$

The former matrix is the (normalized) Fisher information matrix associated with $\mathbf{t}$ and the latter is the Fisher information matrix associated with Lebesgue measure on S_1.

Proof. Since $\mathbf{t}$ is equispaced Lemma S1-1.22 can be applied to each entry of the integrand and the result follows. □

The preceding lemmas allow us to obtain optimality of equispaced designs for power optimization as a corollary of Theorem S1-1.18.

Corollary S1-1.23.1. *Designs with measurements equispaced along all frequencies of interest with $N \geq 4$ measurements provide optimal worst-case power.*

Proof. Lemmas S1-1.20 and S1-1.21 verify that Theorem S1-1.18 applies to power optimization, so Lebesgue measure provides a global lower bound on the problem. By Lemma S1-1.23, equispaced designs have the same Fisher information matrix as Lebesgue measure and therefore achieve the global bound. □

S1-1.7 Period uncertainty and multiple test correction

The optimal designs presented in Sect 3.2 were obtained by maximizing the worst-case fixed-period power across all frequencies of interest. Since the data produced from such a design would be analyzed under period uncertainty, we provide a brief discussion of how the fixed-period power relates to the actual power of a rhythm detection study with discrete period uncertainty.

Suppose the study investigates frequencies $\{f_1, \ldots, f_m\}$ by computing a p-value from an F-test (Definition 2.1) for each frequency and quantifying significance using Bonferonni correction. In this case, the worst-case power can be interpreted as a lower bound on the true power of the test. Indeed, since we have the inclusion

$$\left\{\mathbf{x} \in \mathbb{R}^n : p_i(\mathbf{x}; \boldsymbol{\beta}, \mathbf{t}) \leq \frac{\alpha}{m}\right\} \subset \left\{\mathbf{x} \in \mathbb{R}^n : \min_{1 \leq i \leq m} p_i(\mathbf{x}; \boldsymbol{\beta}, \mathbf{t}) \leq \frac{\alpha}{m}\right\}, \tag{S104}$$

in which $p_i(\mathbf{x}; \mathbf{t})$ is the p-value corresponding to frequency $f_i \in \{f_1, \ldots, f_m\}$. A relation between the fixed-period power at each frequency and the true power of the Bonferonni-corrected test follows immediately from Eq S104 and the definition of power given in Eq 5,

$$\gamma_i(\mathbf{t}; \boldsymbol{\beta}, \alpha/m) = \mathbb{P}_{\boldsymbol{\beta}}\left(p_i(\mathbf{X}; \mathbf{t}) \leq \frac{\alpha}{m}\right) \leq \mathbb{P}_{\boldsymbol{\beta}}\left(\min_{1 \leq i \leq m} p_i(\mathbf{X}; \mathbf{t}) \leq \frac{\alpha}{m}\right) = \Gamma_b(\mathbf{t}; \boldsymbol{\beta}, f_1, \ldots, f_m), \tag{S105}$$

in which γ_i is the power of the cosinor model at frequency f_i, $\boldsymbol{\beta}$ is a parameter vector, and Γ_b is the power function for the Bonferonni-corrected test.

S1-2 Derivation of permutation power bound

S1-2.1 Moments of quadratic forms

We use the notation π to refer to the permutation $\pi \in S_n$ and its matrix representation $\phi(\pi) \in \mathbb{M}_n(\mathbb{R})$ interchangeably. Given a multi-index $\boldsymbol{\alpha} = (\alpha_i)_{i=1}^d \in \{1, \ldots, n\}^d$, the notation $\hat{\boldsymbol{\alpha}}$ refers to the function $\hat{\boldsymbol{\alpha}} : \{1, \ldots, d\} \to \{1, \ldots, n\}$ given by $\hat{\boldsymbol{\alpha}}(i) = \alpha_i$.

Permutation tests involve averaging a quadratic form over the action of the permutation group. To estimate the power of such a test, it will be useful to have simple expressions for moments computed either with respect to the argument of the quadratic form (i.e. $\mathbb{E}_{\mathbf{x}}[\mathbf{x}^T Q \mathbf{x}]$)), or with respect to the action of a matrix group (i.e. $\mathbb{E}_{\pi}[\mathbf{x}^T \pi^T Q \pi \mathbf{x}]$). We collect these exact expressions below and provide brief derivations as needed.

Lemma S1-2.1 (Mean of quadratic form)**.** *Let* $\mathbf{x}$ *be a random vector with mean* $\boldsymbol{\mu}$ *and covariance matrix* Σ*. The mean of the quadratic form* $T(\mathbf{x}) = \mathbf{x}^T Q \mathbf{x}$ *with respect to* $\mathbf{x}$ *is given by*

$$\mathbb{E}_{\mathbf{x}}[T(\mathbf{x})] = \operatorname{tr}(Q\Sigma) + \boldsymbol{\mu}^T Q \boldsymbol{\mu}. \tag{S106}$$

Proof. The result follows from the linearity and cyclicity of trace

$$\mathbb{E}_{\mathbf{x}}[T(\mathbf{x})] = \mathbb{E}_{\mathbf{x}}[\operatorname{tr}\left(\mathbf{x}^T Q \mathbf{x}\right) = \operatorname{tr}\left(Q\mathbb{E}[\mathbf{x}\mathbf{x}^T]\right) = \operatorname{tr}\left(Q(\Sigma + \boldsymbol{\mu}\boldsymbol{\mu}^T)\right) = \operatorname{tr}(Q\Sigma) + \boldsymbol{\mu}^T Q \boldsymbol{\mu}. \tag{S107}$$

□

Expectations of the form

$$\mathbb{E}_{\pi}[\mathbf{x}^T \pi^T Q \pi \mathbf{x}] = \frac{1}{n!} \sum_{\pi \in S_n} \left(\mathbf{x}^T \pi^T Q \pi \mathbf{x}\right)^k, \tag{S108}$$

can be computed exactly using simple combinatorial techniques reviewed in [9] and summarized in Theorem S1-2.2. The essential idea is that each matrix element in Eq S108 can be computed by evaluating sums of the form $\sum_{\pi \in S_n} \left[\prod_{i=1}^d \pi_{\alpha_i \beta_j}\right]$ for suitably chosen multi-indices $\boldsymbol{\alpha}$ and $\boldsymbol{\beta}$. Importantly, these sums can only be nonzero if $\boldsymbol{\alpha}$ and $\boldsymbol{\beta}$ have the same structure in their repeated indices. This structure is known as combinatorial type, which we define below.

Definition S1-2.1 (Combinatorial type)**.** Let $\boldsymbol{\alpha}, \boldsymbol{\beta} \in \{1, \ldots, n\}^d$ be multi-indices and consider the associated

maps $\hat{\boldsymbol{\alpha}}, \hat{\boldsymbol{\beta}} : \{1, \ldots, d\} \to \{1, \ldots, n\}$ given by

$$\hat{\boldsymbol{\alpha}}(i) = \alpha_i, \quad \hat{\boldsymbol{\beta}}(i) = \beta_i. \tag{S109}$$

If the fibers of $\hat{\boldsymbol{\alpha}}$ and $\hat{\boldsymbol{\beta}}$ induce the same partition on their domain, then $\boldsymbol{\alpha}$ and $\boldsymbol{\beta}$ are said to be of the same combinatorial type.

For example, the following multi indices all induce the same partition on their domain $\{1, 2, 3, 4\}$ and are therefore of the same type

$$\text{type}([1166]) = \text{type}([2266]) = \text{type}([3388]), \tag{S110}$$

whereas $\text{type}([1616]) \neq \text{type}([1166])$ since their fibers induce distinct partitions.

Definition S1-2.2 (Type size)**.** For a multi-index $\boldsymbol{\alpha} \in \{1, \ldots, n\}^d$ and associated map $\hat{\boldsymbol{\alpha}} : \{1, \ldots, d\} \to \{1, \ldots, n\}$ the type size of $\boldsymbol{\alpha}$, denoted by $\#\text{type}(\boldsymbol{\alpha})$, is equal to the number of nonempty fibers of $\hat{\boldsymbol{\alpha}}$.

Equipped with these definitions we can state the main theorem which will be used repeatedly throughout our derivation of power bounds.

Theorem S1-2.2 (Expectation of permutation matrix elements, [9])**.** *Let $f(\cdot; \boldsymbol{\alpha}, \boldsymbol{\beta}) : S_n \to \mathbb{R}$ be a function of the form*

$$f(\pi; \boldsymbol{\alpha}, \boldsymbol{\beta}) = \prod_{i=1}^{d} \pi_{\alpha_i \beta_i}. \tag{S111}$$

The expectation $\mathbb{E}_\pi[f(\pi; \boldsymbol{\alpha})]$ with respect to Haar measure on the permutation group

$$\mathbb{E}_\pi[f(\pi; \boldsymbol{\alpha}, \boldsymbol{\beta})] = \frac{1}{n!} \sum_{\pi \in S_n} f(\pi; \boldsymbol{\alpha}, \boldsymbol{\beta}) \tag{S112}$$

is given by

$$\mathbb{E}_\pi[f(\pi; \boldsymbol{\alpha}, \boldsymbol{\beta})] = \delta_{\text{type}(\boldsymbol{\alpha})\text{type}(\boldsymbol{\beta})} \frac{(n - \#\text{type}(\boldsymbol{\alpha}))!}{n!} \tag{S113}$$

In the following two corollaries, we use Theorem S1-2.2 to evaluate Eq S108 for $k = 1$ and $k = 2$.

Corollary S1-2.2.1 (Expectation of quadratic form over permutation group)**.** *Let $Q \in M_n(\mathbb{R})$ be symmetric and*

consider the quadratic form $T(\mathbf{x}) = \mathbf{x}^T Q\mathbf{x}$. *For a fixed* $\mathbf{x} \in \mathbb{R}^n$, *the average of this quadratic form is given by*

$$\mathbb{E}_\pi[T(\pi\mathbf{x})] = w_1 \operatorname{tr}(Q)\, \mathbf{x}^T\mathbf{x} + w_2 \operatorname{tr}(QJ)\, \mathbf{x}^T J\mathbf{x} \tag{S114}$$

in which $w_k = \frac{(n-k)!}{n!}$ *and* $J_{ij} = 1 - \delta_{ij}$.

Proof. Use linearity of expectation to write

$$\mathbb{E}_\pi[T(\pi\mathbf{x})] = \sum_{ijk\ell} \mathbb{E}_\pi[\pi_{ij}x_j L_{ik}\pi_{k\ell}x_\ell] = \sum_{ijk\ell} L_{ik}\mathbb{E}_\pi[\pi_{ij}\pi_{k\ell}]x_j x_\ell. \tag{S115}$$

By Theorem S1-2.2 we can reduce the expectation value in Eq S115 to a sum over multi-index types. Since there are exactly two combinatorial types for multi-indices of length two (either $\alpha_1 = \alpha_2$ or $\alpha_1 \neq \alpha_2$ for $\boldsymbol{\alpha} = (\alpha_1, \alpha_2)$), the expectation reduces to

$$\mathbb{E}_\pi[\pi_{ij}\pi_{k\ell}] = \delta_{ik}\delta_{j\ell}w_1 + (1 - \delta_{ik}\delta_{j\ell})w_2 \tag{S116}$$

in which $w_k = (n-k)!/n!$ and Eq S115 becomes

$$E_\pi[T(\pi\mathbf{x})] = \sum_{ijk\ell} L_{ik} \left(\delta_{ik}\delta_{j\ell}w_1 + (1 - \delta_{ik}\delta_{j\ell})w_2\right) x_j x_\ell \tag{S117}$$

$$= \sum_i L_{ii} \sum_j x_j^2 w_1 + w_2 \sum_{ikj\ell} L_{ik}(1 - \delta_{ik})x_j(1 - \delta_{j\ell})x_\ell \tag{S118}$$

$$= \operatorname{tr}(L)\, \mathbf{x}^T\mathbf{x}w_1 + \operatorname{tr}(LJ)\, \mathbf{x}^T J\mathbf{x}w_2. \tag{S119}$$

□

Corollary S1-2.2.2 (Second moment of quadratic form over permutation group). *For* $n \geq 4$ *let* $W_{ik\alpha\gamma}^{j\ell\beta\rho}$ *be the tensor given by*

$$W_{ik\alpha\gamma}^{j\ell\beta\rho} = \delta_{\text{type}([i\ k\ \alpha\ \gamma])\text{type}([j\ \ell\ \beta\ \rho])} \frac{(n - \#\text{type}([i\ k\ \alpha\ \gamma]))!}{n!}. \tag{S120}$$

Let $Q \in M_n(\mathbb{R})$ *be symmetric and consider the quadratic form* $T(\mathbf{x}) = \mathbf{x}^T Q\mathbf{x}$. *For a fixed* $\mathbf{x} \in \mathbb{R}^n$, *the second moment of* $T(\pi\mathbf{x})$ *with respect to the uniform measure on the permutation group is given by*

$$\mathbb{E}_\pi[T(\pi\mathbf{x})^2] = \sum_{ijkl} \sum_{\alpha\beta\gamma\rho} Q_{ik}Q_{\alpha\gamma}W_{ik\alpha\gamma}^{j\ell\beta\rho}x_j x_\ell x_\beta x_\rho \tag{S121}$$

The tensor W contains nonzero entries corresponding to the 15 equivalence classes of combinatorial types for multi-indices $\boldsymbol{\alpha} \in \{1, \ldots, n\}^4$ with $n \geq 4$.

Proof. Expand using linearity to obtain

$$\mathbb{E}_\pi[T(\pi \mathbf{x})^2] = \sum_{ijkl} \sum_{\alpha\beta\gamma\rho} Q_{ik} Q_{\alpha\gamma} \mathbb{E}_\pi[\pi_{ij}\pi_{kl}\pi_{\alpha\beta}\pi_{\gamma\rho}] x_j x_\ell x_\beta x_\rho = \sum_{ijkl} \sum_{\alpha\beta\gamma\rho} Q_{ik} Q_{\alpha\gamma} W^{j\ell\beta\rho}_{ik\alpha\gamma} x_j x_\ell x_\beta x_\rho. \tag{S122}$$

Theorem S1-2.2 reduces Eq S122 to a sum over combinatorial types. The diagrams below show the 15 combinatorial types for multi-indices of length 4. The diagrams are arranged into families based on the number and sizes of non-empty fibers.

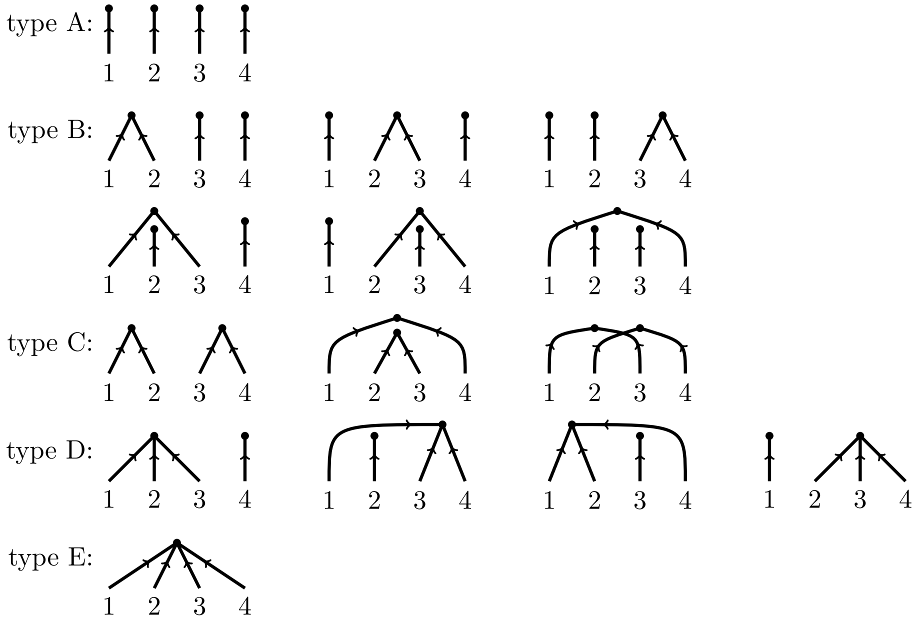

(S123)

(S124)

(S125)

(S126)

(S127)

(S128)

□

Since the tensor W is constant for entries of each type, the expectation in Eq S121 can be computed by evaluating the contribution of each combinatorial type individually. We use the notation $E_A[T(\mathbf{x})], E_B[T(\mathbf{x})], \ldots, E_E[T(\mathbf{x})]$ to denote the contribution of each combinatorial type to the expectation and compute these terms below.

Lemma S1-2.3 (Type A). *The Type A contribution is given by*

$$E_A[T(\mathbf{x})] = \frac{(n-4)!}{n!}\Big(\operatorname{tr}\left(\left(J(J\odot Q)J + Q\right)(J\odot Q)\right) - 2\mathbf{1}^T(J\odot Q)(J\odot Q)\mathbf{1}\Big)$$
$$\times\Big(\operatorname{tr}\left(\left(J\left(J\odot \mathbf{x}\mathbf{x}^T\right)J + \mathbf{x}\mathbf{x}^T\right)\left(J\odot \mathbf{x}\mathbf{x}^T\right)\right) - 2\mathbf{1}^T\left(J\odot \mathbf{x}\mathbf{x}^T\right)\left(J\odot \mathbf{x}\mathbf{x}^T\right)\mathbf{1}\Big), \tag{S129}$$

in which $\odot$ is the Hadamard product and $J \in \mathbb{R}^{n\times n}$ is given by $J_{ij} = 1 - \delta_{ij}$.

Proof. Type A terms require all entries to be distinct, so we may write

$$E_A[T(\mathbf{x})] = \sum_{\substack{\text{type}([ik\alpha\gamma])=A \\ \text{type}([j\ell\beta\rho])=A}} Q_{ik}Q_{\alpha\gamma}W^{j\ell\beta\rho}_{ik\alpha\gamma}x_jx_\ell x_\beta x_\rho \tag{S130}$$

$$= \frac{(n-4)!}{n!}\left(\sum_{ik\alpha\gamma} Q_{ik}Q_{\alpha\gamma}J_{ik}J_{k\alpha}J_{\alpha\gamma}J_{\gamma i}J_{i\alpha}J_{k\gamma}\right)\left(\sum_{j\ell\beta\rho} x_jx_\ell x_\beta x_\rho J_{j\ell}J_{\ell\beta}J_{\beta\rho}J_{\rho j}J_{j\beta}J_{\ell\rho}\right), \tag{S131}$$

with $J_{ij} = 1 - \delta_{ij}$. The first term in parentheses from Eq S130 can be rewritten as

$$\sum_{ik\alpha\gamma} Q_{ik}Q_{\alpha\gamma}J_{ik}J_{k\alpha}J_{\alpha\gamma}J_{\gamma i}J_{i\alpha}J_{k\gamma} = \sum_{ik\alpha\gamma} Q_{ik}Q_{\alpha\gamma}J_{ik}\left(1-\delta_{k\alpha}\right)J_{\alpha\gamma}\left(1-\delta_{\gamma i}\right)J_{i\alpha}J_{k\gamma} \tag{S132}$$

$$= \sum_{ik\alpha\gamma} Q_{ik}Q_{\alpha\gamma}J_{\alpha\gamma}J_{\gamma i}J_{i\alpha}J_{k\gamma}\left(1-\delta_{k\alpha}-\delta_{\gamma i}+\delta_{k\alpha}\delta_{\gamma i}\right). \tag{S133}$$

The terms in Eq S132 simplify to

$$\sum_{ik\alpha\gamma} J_{\alpha\gamma}J_{\gamma i}J_{i\alpha}J_{k\gamma} = \operatorname{tr}\left(J(J\odot Q)J(J\odot Q)\right), \tag{S134}$$

$$\sum_{ik\alpha\gamma} \delta_{k\alpha}J_{\alpha\gamma}J_{\gamma i}J_{i\alpha}J_{k\gamma} = \mathbf{1}^T(J\odot Q)(J\odot Q)\mathbf{1}, \tag{S135}$$

$$\sum_{ik\alpha\gamma} \delta_{\gamma i}J_{\alpha\gamma}J_{\gamma i}J_{i\alpha}J_{k\gamma} = \mathbf{1}^T(J\odot Q)(J\odot Q)\mathbf{1}, \tag{S136}$$

$$\sum_{ik\alpha\gamma} \delta_{k\alpha}\delta_{\gamma i}J_{\alpha\gamma}J_{\gamma i}J_{i\alpha}J_{k\gamma} = \operatorname{tr}\left((J\odot Q)Q\right), \tag{S137}$$

and after collecting like terms we obtain

$$\sum_{ik\alpha\gamma} Q_{ik}Q_{\alpha\gamma}J_{ik}J_{k\alpha}J_{\alpha\gamma}J_{\gamma i}J_{i\alpha}J_{k\gamma} = \operatorname{tr}\left(\left(J(J\odot Q)J + Q\right)(J\odot Q)\right) - 2\mathbf{1}^T(J\odot Q)(J\odot Q)\mathbf{1}. \tag{S138}$$

The claim follows by applying identical reasoning to the second term in parentheses from Eq S132. □

Lemma S1-2.4 (Type B). *The Type B contribution is given by*

$$E_B[T(\mathbf{x})] = \frac{(n-3)!}{n!}\Big(2\,\mathrm{tr}\left(J\,\mathrm{diag}\,QJ(J\odot Q)\right)\mathrm{tr}\left(J\,\mathrm{diag}\,\mathbf{x}\mathbf{x}^T J(J\odot \mathbf{x}\mathbf{x}^T)\right)$$
$$+ 4\,\mathrm{tr}\left(J(J\odot Q)(J\odot Q)\right)\mathrm{tr}\left(J(J\odot \mathbf{x}\mathbf{x}^T)(J\odot \mathbf{x}\mathbf{x}^T)\right)\Big) \quad \text{(S139)}$$

Proof. Six diagrams of Type B contribute to expectation, we evaluate these terms in the order in which they are presented diagrammatically. Focusing on $E_B^{(1)}[T(\mathbf{x})]$, we require the indices $[ik\alpha\gamma]$ to agree on the first two components (δ_{ik}) and require all other indices to be pairwise distinct. Since the same $[j\ell\beta\rho]$ must be of the same combinatorial type, we obtain the following expression

$$E_B^{(1)}[T(\mathbf{x})] = \frac{(n-3)!}{n!}\left(\sum_{ik\alpha\gamma} Q_{ik}Q_{\alpha\gamma}\delta_{ik}J_{k\alpha}J_{k\gamma}J_{\alpha\gamma}\right)\left(\sum_{j\ell\beta\rho} x_j x_\ell x_\beta x_\rho \delta_{j\ell}J_{\ell\beta}J_{\ell\rho}J_{\beta\rho}\right). \quad \text{(S140)}$$

The terms in parentheses simplify to

$$\sum_{ik\alpha\gamma} Q_{ik}Q_{\alpha\gamma}\delta_{ik}J_{k\alpha}J_{k\gamma}J_{\alpha\gamma} = \mathrm{tr}\left(J\,\mathrm{diag}\,QJ(J\odot Q)\right), \quad \text{(S141)}$$

$$\sum_{j\ell\beta\rho} x_j x_\ell x_\beta x_\rho \delta_{j\ell}J_{\ell\beta}J_{\ell\rho}J_{\beta\rho} = \mathrm{tr}\left(J\,\mathrm{diag}\,\mathbf{x}\mathbf{x}^T J(J\odot \mathbf{x}\mathbf{x}^T)\right), \quad \text{(S142)}$$

agreeing with the first term in Eq S139. Applying analogous reasoning to $E_B^{(2)}[T(\mathbf{x})]$ gives

$$E_B^{(2)}[T(\mathbf{x})] = \frac{(n-3)!}{n!}\left(\sum_{ik\alpha\gamma} Q_{ik}Q_{\alpha\gamma}J_{ik}\delta_{k\alpha}J_{k\gamma}J_{\alpha\gamma}\right)\left(\sum_{j\ell\beta\rho} Q_{j\ell}Q_{\beta\rho}J_{j\ell}\delta_{\ell\beta}J_{\ell\rho}J_{\beta\rho}\right) \quad \text{(S143)}$$

$$= \mathrm{tr}\left(J(J\odot Q)(J\odot Q)\right)\mathrm{tr}\left(J(J\odot \mathbf{x}\mathbf{x}^T)(J\odot \mathbf{x}\mathbf{x}^T)\right). \quad \text{(S144)}$$

Each of the remaining terms agree with the two terms already computed. In particular, $E_B^{(3)}[T(\mathbf{x})] = E_B^{(1)}[T(\mathbf{x})]$ and all remaining terms coincide with $E_B^{(2)}[T(\mathbf{x})]$. Adding the terms together we obtain the expected result

$$E_B[T(\mathbf{x})] = \sum_{r=1}^{6} E_B^{(r)}[T(\mathbf{x})] = 2E^{(1)}[T(\mathbf{x})] + 4E^{(2)}[T(\mathbf{x})]. \quad \text{(S145)}$$

□

Lemma S1-2.5 (Type C). *The Type C contribution is given by*

$$E_C[T(\mathbf{x})] = \frac{(n-2)!}{n!}\Big(\big(\mathbf{1}^T \operatorname{diag} QJ \operatorname{diag} Q\mathbf{1}\big)\left(\mathbf{1}^T \operatorname{diag} \mathbf{x}\mathbf{x}^T J \operatorname{diag} \mathbf{x}\mathbf{x}^T \mathbf{1}\right)$$
$$+ 2\operatorname{tr}((J\odot Q)Q)\operatorname{tr}\left((J\odot \mathbf{x}\mathbf{x}^T)\mathbf{x}\mathbf{x}^T\right)\Big) \tag{S146}$$

Proof. There are three diagrams of Type C, the first diagram simplifies to

$$E_C^{(1)}[T(\mathbf{x})] = \frac{(n-2)!}{n!}\left(\sum_{ik\alpha\gamma} Q_{ik}Q_{\alpha\gamma}\delta_{ik}\delta_{\alpha\gamma}J_{k\alpha}\right)\left(\sum_{j\ell\beta\rho} x_j x_\ell x_\beta x_\rho \delta_{j\ell}\delta_{\beta\rho}J_{\ell\beta}\right) \tag{S147}$$
$$= \big(\mathbf{1}^T \operatorname{diag} QJ \operatorname{diag} Q\mathbf{1}\big)\left(\mathbf{1}^T \operatorname{diag} \mathbf{x}\mathbf{x}^T J \operatorname{diag} \mathbf{x}\mathbf{x}^T \mathbf{1}\right), \tag{S148}$$

and the second term simplifies to

$$E_C^{(2)}[T(\mathbf{x})] = \frac{(n-2)!}{n!}\left(\sum_{ik\alpha\gamma} Q_{ik}Q_{\alpha\gamma}\delta_{i\alpha}\delta_{k\gamma}J_{ik}\right)\left(\sum_{j\ell\beta\rho} x_j x_\ell x_\beta x_\rho \delta_{j\beta}\delta_{\ell\rho}J_{j\ell}\right) \tag{S149}$$
$$= \operatorname{tr}((J\odot Q)Q)\operatorname{tr}\left((J\odot \mathbf{x}\mathbf{x}^T)\mathbf{x}\mathbf{x}^T\right). \tag{S150}$$

Finally we find that $E_C^{(3)}[T(\mathbf{x})] = E_C^{(2)}[T(\mathbf{x})]$ and the claim follows. □

Lemma S1-2.6 (Type D). *The type D contribution is given by*

$$E_D[T(\mathbf{x})] = \frac{(n-2)!}{n!} 4\operatorname{tr}(\operatorname{diag} QQJ)\operatorname{tr}\left(\operatorname{diag} \mathbf{x}\mathbf{x}^T\mathbf{x}\mathbf{x}^T J\right) \tag{S151}$$

Proof. All four terms of Type D have the same structure so we need only evaluate the first term

$$E_D^{(1)}[T(\mathbf{x})] = \left(\sum_{ik\alpha\gamma} Q_{ik}Q_{\alpha\gamma}\delta_{ik}\delta_{k\alpha}J_{\alpha\gamma}\right)\left(\sum_{j\ell\beta\rho} x_j x_\ell x_\beta x_\rho \delta_{j\ell}\delta_{\ell\beta}J_{\beta\rho}\right) \tag{S152}$$
$$= \operatorname{tr}(\operatorname{diag} QQJ)\operatorname{tr}\left(\operatorname{diag} \mathbf{x}\mathbf{x}^T\mathbf{x}\mathbf{x}\mathbf{x}^T J\right). \tag{S153}$$

□

Lemma S1-2.7 (Type E). *The type E contribution is given by*

$$E_E[T(\mathbf{x})] = \frac{(n-1)!}{n!}\operatorname{tr}(\operatorname{diag} Q \operatorname{diag} Q)\operatorname{tr}\left(\operatorname{diag} \mathbf{x}\mathbf{x}^T \operatorname{diag} \mathbf{x}\mathbf{x}^T\right) \tag{S154}$$

Proof. There is only one term to consider, and we require all indices to coincide

$$E_E[T(\mathbf{x})] = \left(\sum_{ik\alpha\gamma} Q_{ik} Q_{\alpha\gamma} \delta_{ik} \delta_{k\alpha} \delta_{\alpha\gamma}\right) \left(\sum_{j\ell\beta\rho} x_j x_\ell x_\beta x_\rho \delta_{j\ell} \delta_{\ell\beta} \delta_{\beta\rho}\right) \tag{S155}$$

$$= \frac{(n-1)!}{n!} \operatorname{tr}(\operatorname{diag} Q \operatorname{diag} Q) \operatorname{tr}\left(\operatorname{diag} \mathbf{x}\mathbf{x}^T \operatorname{diag} \mathbf{x}\mathbf{x}^T\right). \tag{S156}$$

□

S1-2.2 Permutation power bound

We now use the results of the previous section to bound the power of the permutation test in terms of the moments of its test statistic.

Theorem S1-2.8 (Chebyshev p-value bound)**.** *For a given test statistic $T(\mathbf{x})$ with permutation mean $\mathbb{E}_\pi[T(\mathbf{x})]$ and variance $\mathrm{Var}_\pi[T\pi]$, let $\psi(\mathbf{x};T)$ be the function*

$$\psi(\mathbf{x};T) = \begin{cases} \frac{\mathrm{Var}_\pi[T(\pi\mathbf{x})]}{\mathrm{Var}_\pi[T(\pi\mathbf{x})]+(T_{\mathrm{obs}}-\mathbb{E}_\pi[T(\pi\mathbf{x})])^2} & \text{if } T_{\mathrm{obs}} > \mathbb{E}_\pi[T(\pi\mathbf{x})], \\ 1 & \text{if } T_{\mathrm{obs}} \leq \mathbb{E}_\pi[T(\pi\mathbf{x})], \end{cases} \tag{S157}$$

then the p-value of the permutation test associated to $T(\mathbf{x})$ satisfies

$$P_\pi(T(\pi\mathbf{x}) \geq T_{\mathrm{obs}}) \leq \psi(\mathbf{x};T). \tag{S158}$$

Proof. Recall that for any real-valued random variable X with mean $\mathbb{E}[X]$ and variance σ^2 and any $c > 0$, the one-sided Chebyshev inequality states that

$$P(X - \mathbb{E}[X] > c) \leq \frac{\sigma^2}{\sigma^2 + c^2}. \tag{S159}$$

If $T_{\mathrm{obs}} > \mathbb{E}_\pi[T(\pi\mathbf{x})]$, the first bound follows immediately from applying Eq S159 to $P_\pi(T(\pi\mathbf{x}) \geq T_{\mathrm{obs}})$. If $T_{\mathrm{obs}} \leq \mathbb{E}_\pi[T(\pi\mathbf{x})]$, Chebyshev's inequality no longer applies and we are left with the trivial bound $P_\pi(T(\pi\mathbf{x}) \geq T_{\mathrm{obs}}) \leq 1$. □

Theorem S1-2.9 (Chebyshev power bound)**.** *For a permutation test based on the test statistic $T_2(\mathbf{x})$ given in*

Eq 27, the power γ is bounded below by

$$\gamma = P_{\mathbf{x}}\left(P_\pi(T(\pi\mathbf{x}) \geq T_{\text{obs}}) < \alpha\right) \geq \begin{cases} 1 - \frac{\text{Var}_{\mathbf{x}}[\psi(\mathbf{x};T)]}{\text{Var}_{\mathbf{x}}[\psi(\mathbf{x};T)] + (\alpha - \mathbb{E}_{\mathbf{x}}[\psi(\mathbf{x};T)])^2} & \textit{if } \mathbb{E}_{\mathbf{x}}[\psi(\mathbf{x};T)] < \alpha, \\ 0 & \textit{if } \mathbb{E}_{\mathbf{x}}[\psi(\mathbf{x};T)] \geq \alpha, \end{cases} \tag{S160}$$

where $\psi(\mathbf{x};T)$ is as given in Eq S157.

Proof. Clearly we have

$$P_{\mathbf{x}}\left(P_\pi(T(\pi\mathbf{x}) \geq T_{\text{obs}}) < \alpha\right) \geq P_{\mathbf{x}}\left(\psi(\mathbf{x};T) < \alpha\right) = 1 - P_{\mathbf{x}}\left(\psi(\mathbf{x};T) > \alpha\right) \tag{S161}$$

and the claim follows by applying the one sided Chebyshev inequality to $P_{\mathbf{x}}\left(\psi(\mathbf{x};T) > \alpha\right)$ under the same conditions as the previous proof. □

References

1. Cochran WG. The distribution of quadratic forms in a normal system, with applications to the analysis of covariance. In: Math Proc Camb Phil Soc. vol. 30. Cambridge University Press; 1934. p. 178-91.
2. Searle SR. Linear models. vol. 65. John Wiley & Sons; 1997.
3. Johnson NL, Kotz S, Balakrishnan N. Continuous univariate distributions, Volume 2. John Wiley & Sons; 1995.
4. Abramowitz M, Stegun IA. Handbook of mathematical functions with formulas, graphs, and mathematical tables. National Bureau of Standards; 1964.
5. Fedorov V. Optimal experimental design. WIREs Comput Stats. 2010;2(5):581-9.
6. Pukelsheim F. Optimal design of experiments. SIAM; 2006.
7. Horn RA, Johnson CR. Matrix analysis. 2nd ed. Cambridge university press; 2012.
8. Folland GB. Real analysis: modern techniques and their applications. 2nd ed. John Wiley & Sons; 1999.
9. Collins B, Matsumoto S, Novak J. The Weingarten calculus. Notices Amer Math Soc. 2022;69(05):1.
