# Supplementary figures and images for: Optimization of experimental designs for biological rhythm discovery
Source: PLoS Comput Biol. 2025 Nov 10;21(11):e1013662. doi: 10.1371/journal.pcbi.1013662 (PMC12617917; doi:10.1371/journal.pcbi.1013662)

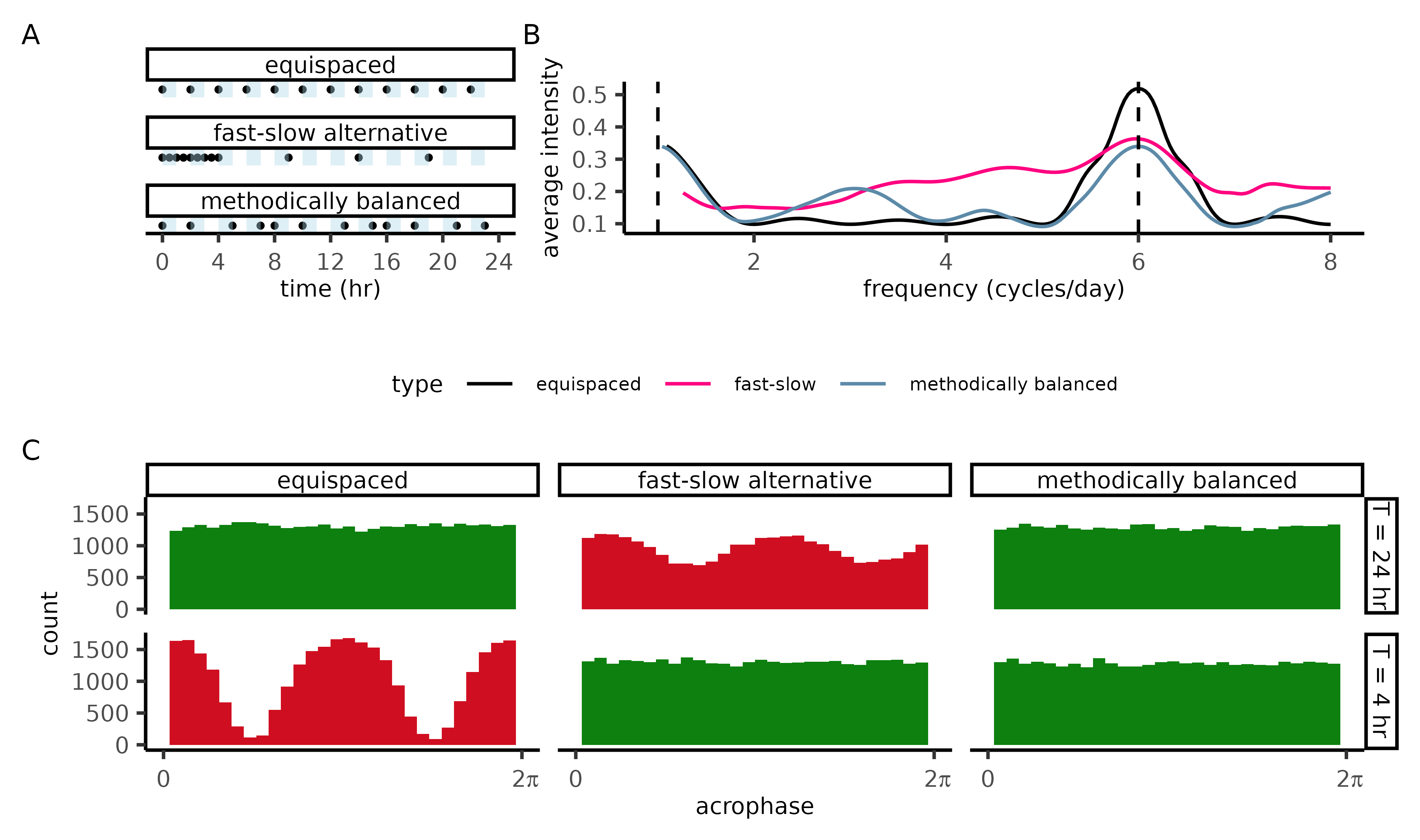

Supplement: S1 Fig — Each oscillator (n = 105) in the simulated dataset was assigned a 24hr or 4hr period and a uniformly random acrophase ϕ∈[0,2π). Measurements are simulated with Gaussian white noise at each measurement time. (A) Measurement schedules for (top) a traditional equispaced design, a (middle) fast-slow irregular design, and (bottom) a methodically constructed irregular design. The shaded bars represent 2hr increments and dots indicate sample collection (N = 12 samples for each design). (B) The average intensity of a Lomb-Scargle periodogram for each design. The true periods in the system are marked by the dashed vertical lines. (C) True acrophases of statistically significant oscillators (p < 0.05) detected by cosinor analysis at each of the true periods. Distributions with phase-dependent detection are shown in red to emphasize that the distribution’s variability is due to a statistical artifact. Simulation parameters: amplitude A=2, noise strength σ=1, acrophase ϕ∼Unif(0,2π), period T=4hr,24hr. The methodically-constructed design was generated using mixed-integer conic programming in PowerCHORD. (TIFF) [file pcbi.1013662.s002.tif]

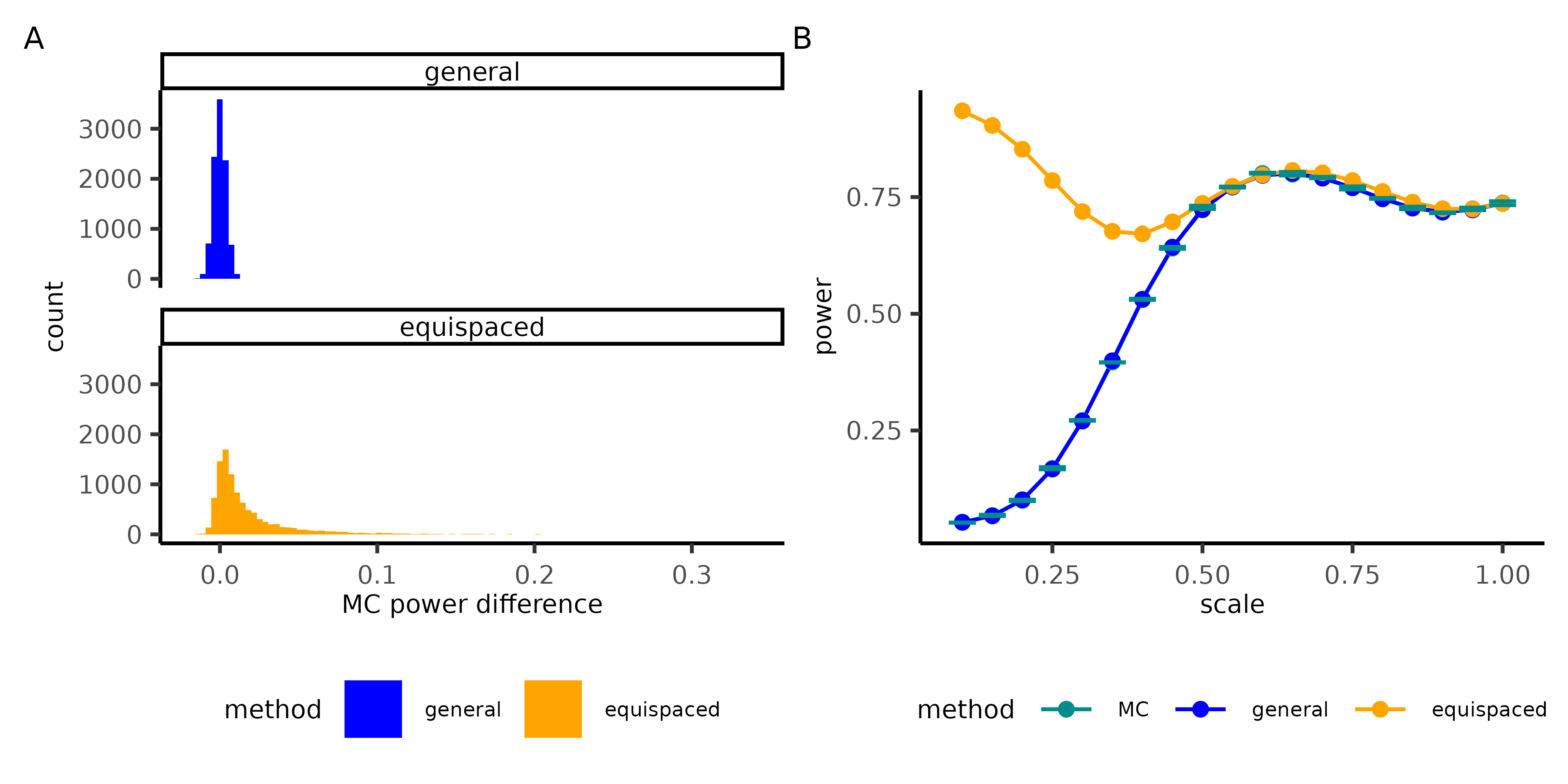

Supplement: S2 Fig — (A) Comparison of our power formula and the equispaced formula to Monte Carlo estimates for randomly generated designs in which t∼unif([0,1]) for each measurement time t. On average, the equispaced formula tended to over-estimate the power of such designs. Parameters: sample size N = 8, amplitude A = 2, frequency f = 1, acrophase ϕ=π, noise strength σ=1. (B) We computed the power of designs 𝐭N,κ=κ𝐭N, where 𝐭N is an N measurement equispaced design and κ>0 is a scale factor. As κ shrinks, the irregularity in the design becomes more pronounced and the equispaced formula diverges from the Monte Carlo power estimates. Parameters: N = 24, A = 1, f = 1, ϕ=0. (TIFF) [file pcbi.1013662.s003.tif]

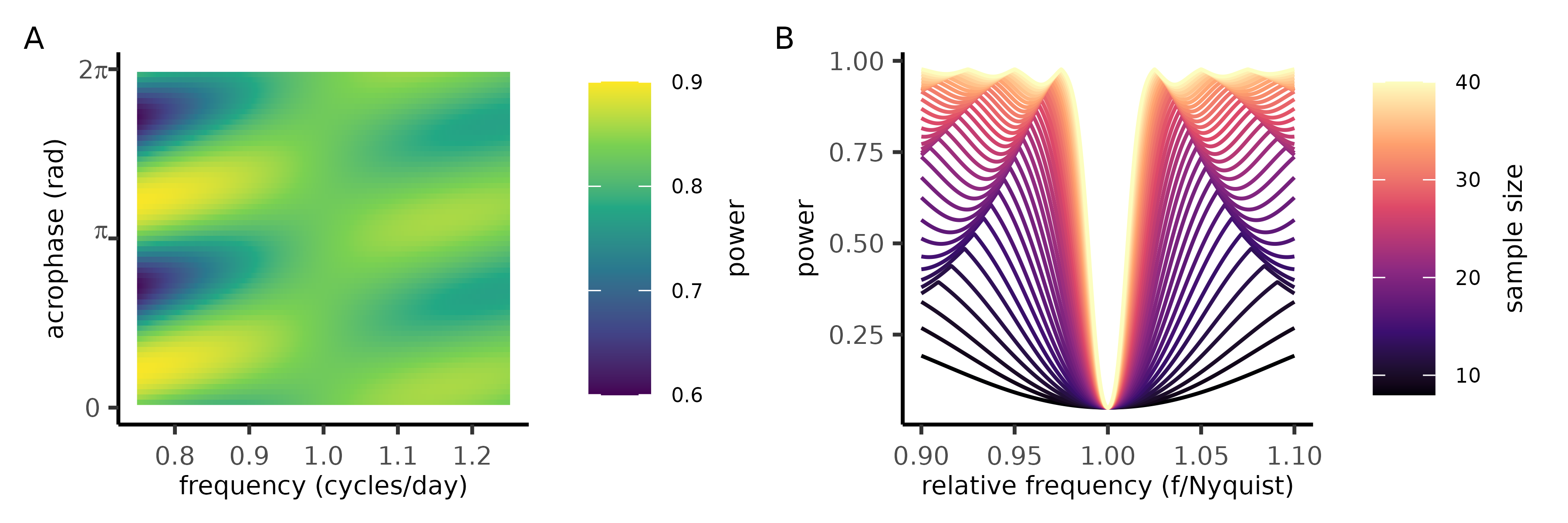

Supplement: S3 Fig — (A) The power (color) of an equispaced design (N = 24 samples) evaluated at each frequency (x-axis) and acrophase (y-axis). The power is independent of phase when the frequency reaches f = 1 because the design is equiphase at this frequency. (B) The worst-case power of equispaced designs (sample size 8≤N≤40) as a function of frequency, with frequency scaled relative to Nyquist rate of each design (frel=f/fNyq). Parameters: amplitude A = 1, noise strength σ=1. (TIFF) [file pcbi.1013662.s004.tif]

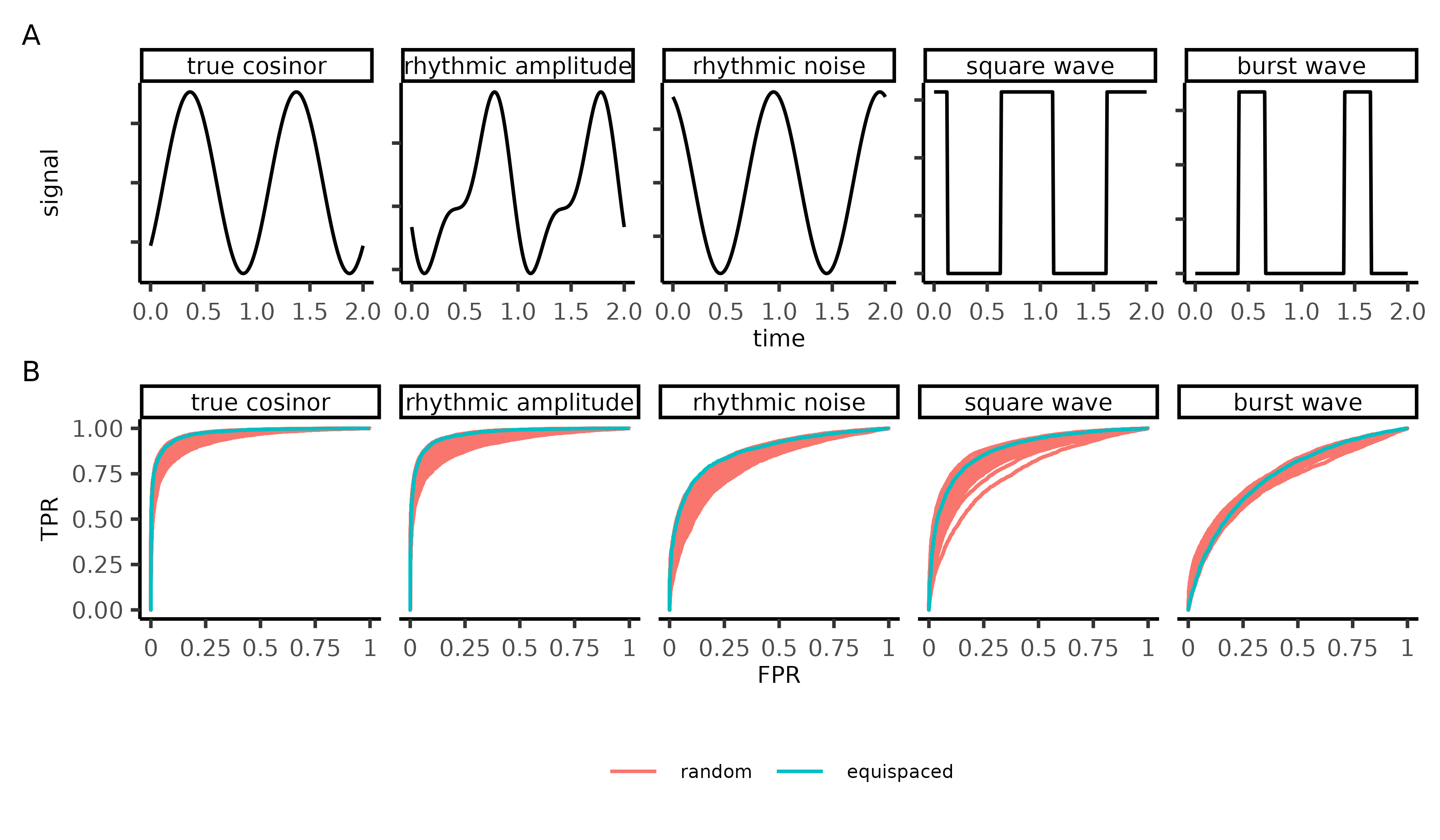

Supplement: S4 Fig — Equispaced designs were compared to randomly generated designs (n = 100) of the same sample size (Nmeas=12) for detecting rhythms that fail to satisfy the assumptions of the cosinor model. (A) Each panel represents a type of signal: (1) standard cosinor, (2) cosinor with amplitude modulation, (3) cosinor with rhythmic noise of the form ε(t)=ε(t)(1+Acos(2πft−ϕ)) where ε(t)∼𝒩(0,1), (4) square wave, and (5) burst-like square wave. For all signal types, the amplitude A and acrophase ϕ vary across simulations, and ε(t)∼𝒩(0,1) represents independent Gaussian noise. (B) True positive rate (y-axis) as a function of false positive rate (x-axis) for each type of signal (panels) and design (color). For each design and signal type, the curves were generated by simulating an ensemble of 5000 white noise signals (null model) and 5000 rhythmic signals (alternative model). Random designs were generated by uniformly random measurement times (t∼Unif([0,1])). Parameters: sample size N = 12, amplitude A∼Unif([1,3]), noise strength σ=1, acrophase ϕ∼Unif([0,2π)), frequency f = 1. Square-like waves were generated with random acrophase and duty cycle 0.5 for square waves and duty cycle 0.25 for burst-waves. For the rhythmic noise model, noise of rhythmic intensity (frequency f = 1) was included in simulations of both the null and alternative models. (TIFF) [file pcbi.1013662.s005.tif]

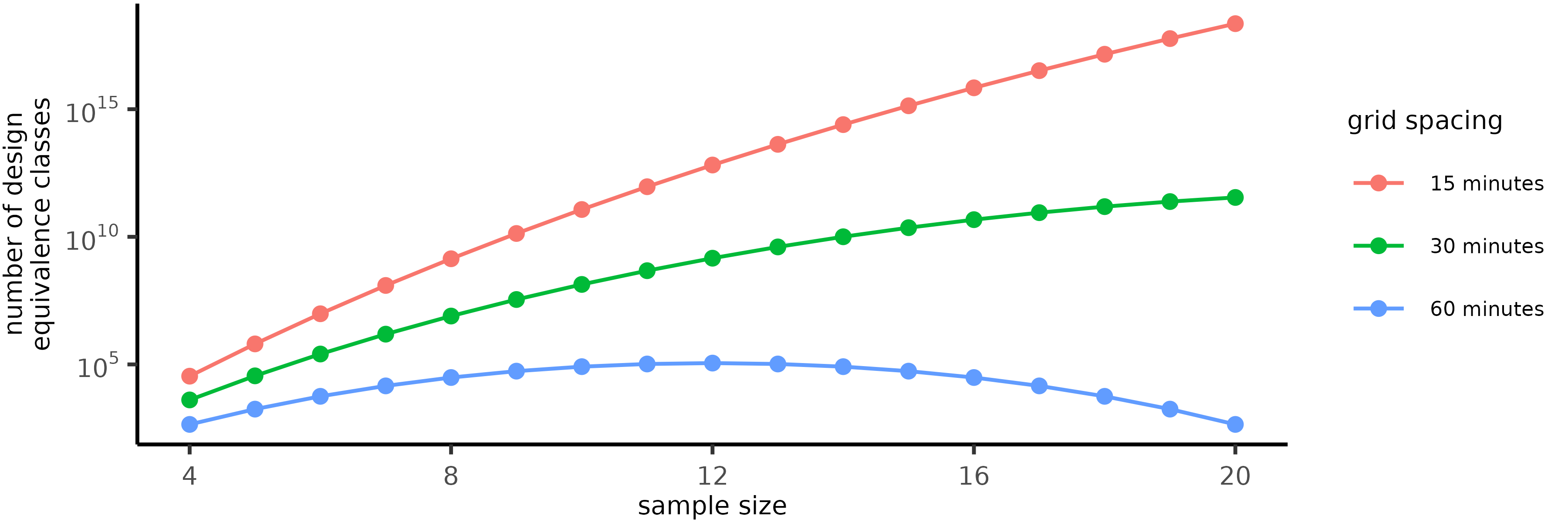

Supplement: S5 Fig — For a given sample size (x-axis) and grid spacing (color), the number of design equivalence classes (y-axis) can be calculated using Eq 31. Designs are in the same equivalence class if they can be transformed into one another by a cyclic shift (i.e. t→t+k/Ntmod1, for some 1≤k≤Nt assuming measurements are in the interval [0,1] and confined to a grid of spacing 1/Nt). (TIFF) [file pcbi.1013662.s006.tif]

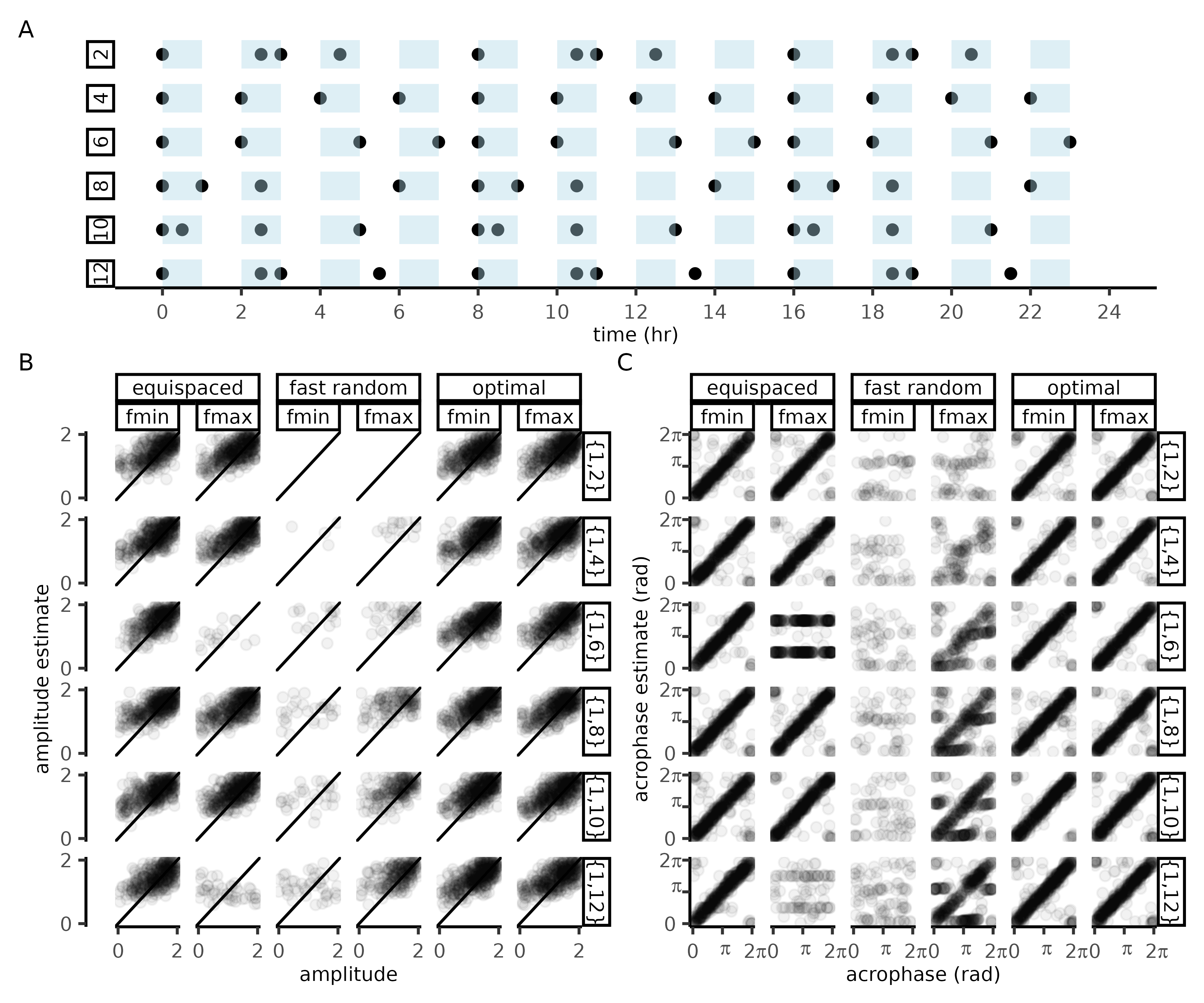

Supplement: S6 Fig — We computed bifrequency optimal designs for frequency priors ν=(1,f) with f∈{2,4,6,8,10,12} and a sample size N = 12. (A) Repetitive patterns appear in the measurement times of the bifrequency optimal designs. (B-C) Comparison of true amplitude and acrophase values to their cosinor estimates after filtering for statistically significance. With the exception of signals at integer multiples of the Nyquist rate (fNyq=6), equispaced and optimal designs performed similarly. At the Nyquist multiples, the optimal designs exhibited much less bias than equispaced designs. A randomly generated design (t∼unif(0,1/12)) with measurements confined to a short timescale was included as a reference. The random design performed poorly at low frequencies and improved as the higher frequency approaches the scale on which its points are distributed. (TIFF) [file pcbi.1013662.s007.tif]

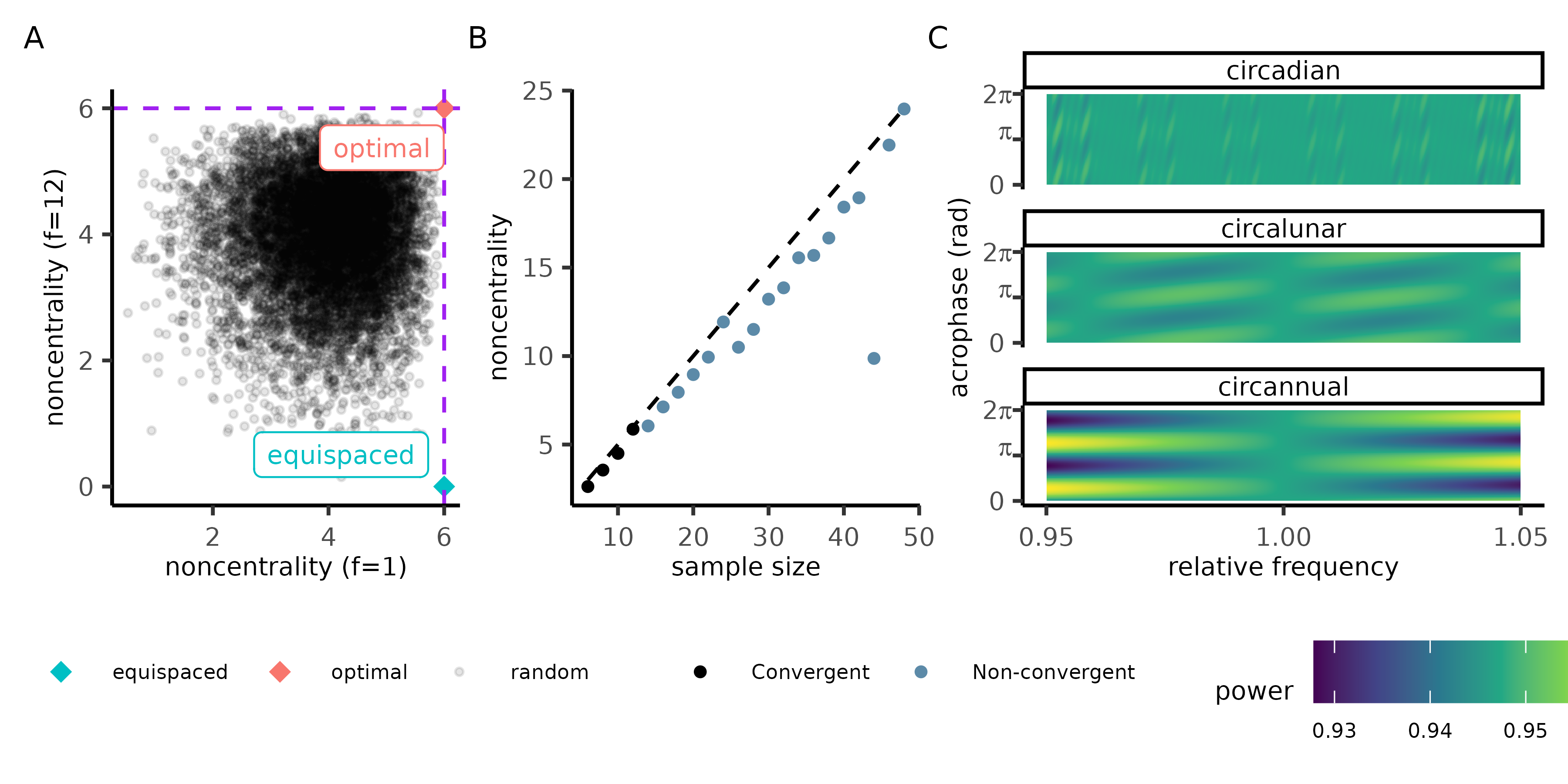

Supplement: S7 Fig — (A) Non-centrality parameters for the two periodicities of interest (f=1 and f=12) for the optimal design (red dot) an equispaced design (blue dot) and an ensemble (n = 104) of randomly generated designs. The theoretical maximum value of the non-centrality parameter (λ=N/2; Theorem 3.2) is indicated by dashed lines. (B) Designs were generated to maximize power at the first N/2 harmonics (f∈(1,…,N/2)) for each sample size N (x-axis). The performance of each design is summarized by the lowest value of its non-centrality parameter (y-axis) across all harmonics included in the optimization. Color indicates convergence of the conic program within 1hr of computation time. The optimal noncentrality parameter in a single frequency design for each sample size (λ=N/2) is shown for reference (dashed line). For sample sizes 1≤N<12 measurements were confined to a 36 point grid, for 12≤N<24 a 48 point grid, and for 24<N a 96 point grid. (C) The trifrequency optimal design with all measurements confined to the first month achieves phase independent power at 24 hr (circadian), 28 day (circalunar), and 12×28=336 day (circannual) periods. Parameters: Amplitude A=1/2. (TIFF) [file pcbi.1013662.s008.tif]

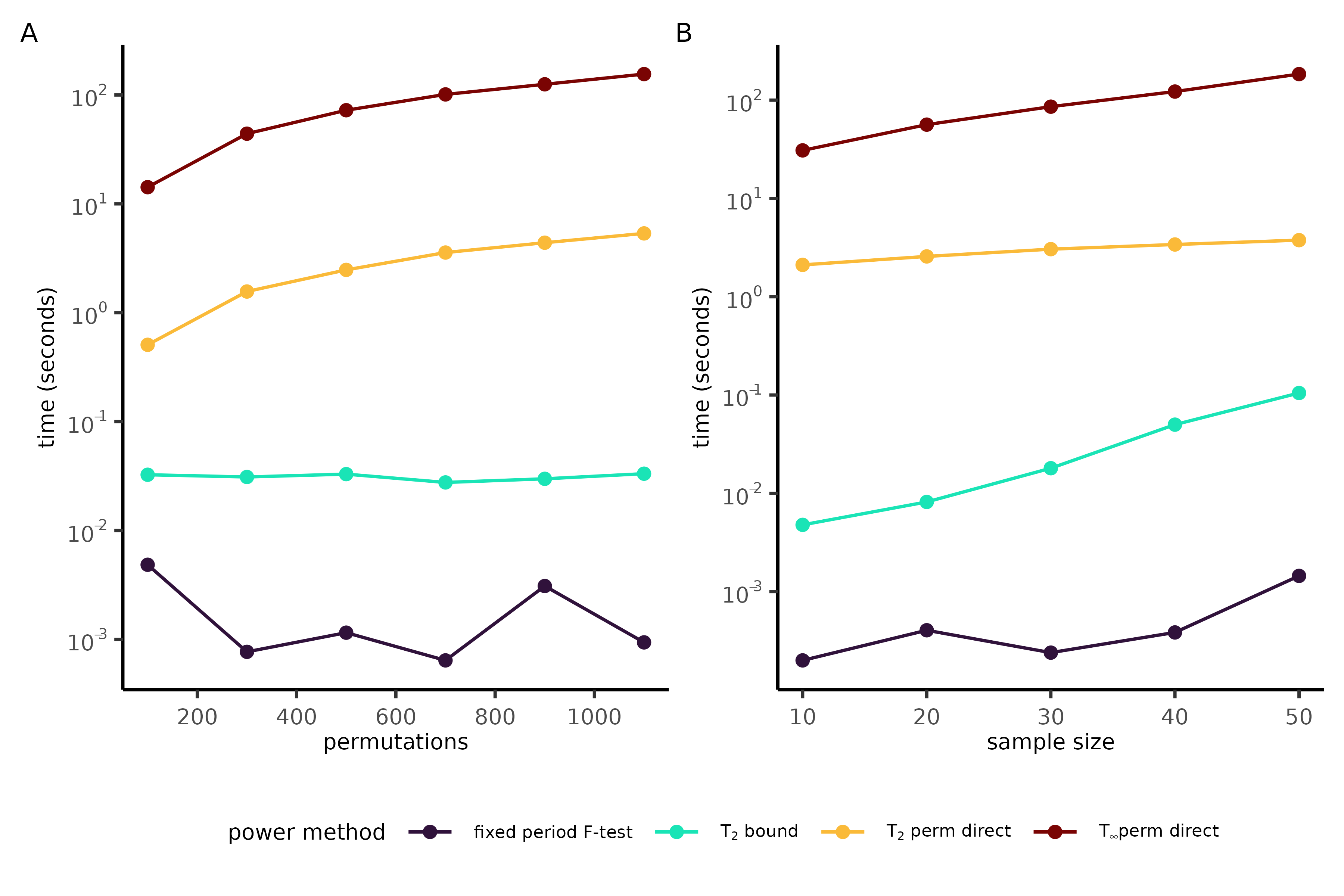

Supplement: S8 Fig — Compute time (y-axis) is shown as a function of (A) the number of permutations (x-axis) and (B) sample size (x-axis) for each power method (color). Parameters: sample size fixed at N = 24 in (A) and permutations fixed at Nperm=103 in (B). For both panels, noise samples Nsamp=103, frequency f∼Unif([0,1]), acrophase ϕ∼Unif([0,2π)), amplitude A∼Unif([0,1]), 𝐭∼Unif([0,1]N),and the T∞ test statistic was discretized using Nf=103 frequencies. (TIFF) [file pcbi.1013662.s009.tif]
