# Supplementary material for: Optimization of experimental designs for biological rhythm discovery
Source: PLoS Comput Biol. 2025 Nov 10;21(11):e1013662. doi: 10.1371/journal.pcbi.1013662 (PMC12617917; doi:10.1371/journal.pcbi.1013662)
Supplement: Attachment — Submitted filename: Power-Chord review.pdf [file pcbi.1013662.s010.pdf]

In the manuscript "*PowerCHORD: constructing optimal experimental designs for biological rhythm discovery",* Silverthorne *et al* introduce a computational framework for optimizing the timing of sample collection in experiments aimed at detecting biological rhythms. Using the cosinor model, the authors derive a closed-form expression for statistical power, which enables them to implement three optimization strategies: differential evolution, brute-force search, and mixed-integer conic programming. These methods are used to design potential experiments with enhanced power for rhythm detection across diverse scenarios, including cases of unknown oscillation period, timing constraints in circadian studies (e.g., sleep windows), and multiple rhythmic processes. The results demonstrate that irregular rather than equispaced measurements can optimize power in situations where the oscillation period is unknown. The manuscript is mathematically rigorous, computationally well-motivated, and addresses an interesting problem in the design of time-series experiments in chronobiological research.

However, there seems to also be significant limitations in the current manuscript, in particular with respect to the strength of the underlying cosinor model and its underlying assumptions, sensitivity to noise parameters, assumptions of homoscedasticity, and the overall readability. Importantly, while this manuscript is suggested to be of use to experimentalists and the design of new experiments, there is no demonstration of implementing the method on real datasets. Therefore while it is a very interesting manuscript, major revisions and more careful discussions on limitations will have to be added before a decision of publication can be arrived at.

Major Concerns:

(1) My major concern is with the use of the cosinor model, which assumes symmetric, stationary and sinusoidal waveforms. While this model is indeed broadly used in the literature, typical usage has been limited to detecting rhythms and periodicity of the rhythms. However, I'm not sure why a cosinor model is appropriate for the more nuanced question that the authors are asking here, i.e., how to space out measurements of the rhythms over time in order to optimize power. It is well known that biological rhythms tend to be non-stationary, with amplitude, time-period and phase velocity varying with time. This has been demonstrated carefully, particularly for the circadian clock which is referred to multiple times in this study. Older studies have demonstrated non-stationarity in activity and body temperature rhythms (**PMID: 15038991**). Recent studies show this even at the molecular level, using careful gene expression measurements (mouse tissues in Tong *et al*; https://doi.org/10.1101/2025.04.03.647044 and mouse cell-lines in Nikhat *et al*; https://doi.org/10.1101/2024.06.30.601408). For instance, Figure 2F in Tong *et al* clearly show that phase velocity in not constant across time. Figure 4D in Nikhat *et al* shows how the phases at 8 and 32 hours post synchronization are quite different, though one might expect them to be identical under a 24-hour cosinor model. Therefore, the authors should carefully discuss why a cosinor model might still be suitable in the light of these experimental datasets.

(2) In Theorem 3.2, the authors prove that if N > 3 equispaced measurements can be made per cycle of a rhythm with known frequency, this design optimizes worst-case power. How strongly does this result depend on the underlying assumptions of the cosinor model (as stated above)? For instance, if the oscillation had a non-varying, fixed 24 hour time-period across cycles, but the phase velocity varied as a function of time, would this result still hold? Intuitively it would seem one would need more closely spaced measurements where phase velocity is faster, and less measurements where velocity is slower.

(3) There has been significant effort in recent times to go beyond the cosinor-type models and model non-stationary rhythms with irregularly sampled data. Some examples are wavelet transforms (**PMIDs: 28569118** and **35610418**) and Gaussian Processes (**PMID: 37769241**) that have been used on circadian rhythm analysis. Indeed, the second article carefully demonstrated that accounting for non-stationarity significantly improves waveform and rhythm detection over methods like cosinor and Lomb-Scargle Periodograms. Therefore, would it be possible to extend the power calculations in this work to these non-stationary models – at least numerically, if not with closed form expressions? To me it seems fundamentally important to account for non-stationarities in the underlying data when the question of measurement spacing is of primary interest.

(4) How strongly are the results of this work dependent on the assumption of homoscedastic noise in Eq. (1)? In reality, as can be observed in all the references above, noise at different time points in biological rhythms does not have equal variance. This is not just a technical issue, but for circadian clocks, also arises from the underlying biology of transcription (Nikhat *et al*; https://doi.org/10.1101/2024.06.30.601408). Could the authors incorporate heteroscedasticity, at least in their simulated datasets and check how well their main results hold?

(5) Related to the previous question, I could not understand how the authors estimated sigma, the s.d. of the Gaussian white noise. This term seems important even in the expression for power in Equations 9 and 10, hence a clearer explanation of how it is estimated would be very helpful. This is especially so because at the time of a power calculation, before the actual data is collected, one would not know how much noise will exist in the data.

(6) Finally, a major limitation of the current manuscript is a lack of application to any experimental dataset. As a result, I found it hard to understand how exactly an experimentalist can use PowerCHORD to set up a new experiment – what would be the input data and data format required to run PowerCHORD, parameters, what needs to be prior knowledge etc. For this algorithm to be practically useful, I would strongly urge the authors to provide a worked out example using an experimental dataset. Adding a flowchart in the main text explaining the main steps to be followed to implement the code would also help improving the readability of the manuscript and ease of practical use. While the code is available on Github, this is also not very easily accessible to the potential user, and neither is the "examples/power_analysis.Rmd" file very easy to understand.

Minor Points:

(1) I found the choice of alphabets in the equations a bit confusing. The lambda in Eq. (9) refers to the non-centrality parameter, but does the lambda in Eq. (12) represent the eigenvalues of the matrix B^-1?

(2) The manuscript in its current form will not be easily accessible to most chronobiology researchers, even those with a more computational background. Given that the results are quite interesting and of potential use to a large community of researchers, I would suggest adding some less technical but more conceptual notes in the text, perhaps as Explanatory Boxes. For instance, some intuitive explanations of the three optimization methods used would significantly enhance readability. Similarly, some intuitive explanations of why irregular designs are better than equispaced designs would be very helpful. Currently the manuscript provides no conceptual intuition and the interesting results are somewhat lost behind the mathematical formalism.
