# Supplementary material for: Optimization of experimental designs for biological rhythm discovery
Source: PLoS Comput Biol. 2025 Nov 10;21(11):e1013662. doi: 10.1371/journal.pcbi.1013662 (PMC12617917; doi:10.1371/journal.pcbi.1013662)
Supplement: Attachment — Submitted filename: response.pdf [file pcbi.1013662.s011.pdf]

Adam Stinchcombe
Department of Mathematics, University of Toronto
40 St. George Street, Rm 6290
Toronto, ON M5S 2E4, Canada
stinch@math.toronto.edu
(416) 946-7193

RE: manuscript PCOMPBIOL-D-25-00228 September 26, 2025

Dear Dr. Robinson-Rechavi,

We thank you and the reviewers for the insightful feedback on our manuscript. After thorough consideration of their comments, we found many opportunities to improve our work. Pursuing these research directions led us to several new results, and we are grateful for their thoughtful questions that inspired us to pursue this line of reasoning. We have made substantial changes to the attached manuscript, specifically focused on:

- **Organization and accessibility of results:** Reviewer #2 was concerned about the accessibility of our work to chronobiologists. To address this concern, we have made substantial revisions to the organization and presentation of our results. We divide our results into three distinct experimental contexts (known period, discrete period uncertainty, and continuous period uncertainty), include only the relevant theory in each experimental section, and order the sections so results with the most immediate experimental relevance appear earlier in the manuscript. We also have updated the title of our manuscript and the overall tone to better reflect the scope of our work.

- **Consideration of free-period model:** Reviewer #1 was concerned that our initial submission optimized worst-case power across different frequencies under the fixed-period assumption, yet did not investigate if this approach agrees with the free-period model. In our revised manuscript, we show numerically that there is strong correlation between the worst-case fixed-period power and the free period power. We also include a rigorous lower bound on the power of the free period model amenable to optimization. We construct a design that optimizes this bound and compare its performance (in terms of free-period power) to our existing designs and found minimal differences.

- **Robustness of model assumptions:** Reviewer #2 expressed several concerns about our use of the cosinor model and the applicability of our results to systems that do not satisfy the model's strict assumptions. Using simulations, we have demonstrated that

equispaced optimality continues to hold for several types of non-sinusoidal rhythms. These simulations led us to a simpler and more general proof of equispaced optimality. Our new proof clarifies that the optimality of equispaced designs is less dependent on the cosinor model's assumptions than suggested by our initial proof. We hope that our extended results will be useful in settings where experimentalists need to determine if they have sufficient prior knowledge to justify deviation from equispaced designs.

Detailed responses to all critiques are provided below, with references to the appropriate sections of the manuscript. We thank you for your re-consideration of our manuscript and we hope that you will find that it suitable for publication in PLOS Computational Biology.

Cheers,

Adam Stinchcombe

Adam Stinchcombe
Associate Professor of Mathematics
on behalf of all co-authors

Reviewer 1

Major points

1. The application proposed here is to design experiments where the period is unknown or partially unknown. When analyzing the data from such an experiment, one must therefore use a cosinor model with an unknown period. However, the power formula given in Theorem 3.1 is for a cosinor model with a fixed and known period. The authors minimize this known-period cosinor power over all periods of interest, but this is not the same as computing the power of the unknown-period cosinor model. The unknown-period model is a non-linear model (at least when the space of periods of interest includes an interval, as is done at points in this manuscript) and has complications such as aliasing (there is no longer a unique least squares or maximum likelihood solution in some cases). It is possible (though I have not verified either way) that these results are therefore only approximations of the true power - and if so, the accuracy of this approximation must be assessed through simulation, etc.. The periodogram Lomb-Scargle analysis is already a step in this direction, but the implications or limitations involved must be addressed explicitly.

Our revised manuscript clarifies the relevance of period uncertainty to our existing results and extends our analysis to include the free period model as needed. We have reorganized our results based on the knowledge of period at the time of study design (Sec. 3.1: known period, Sec. 3.2: discrete period uncertainty, and Sec. 3.3: continuous period uncertainty). For the known-period case, we analyze the fixed-period cosinor model as is standard in the absence of period uncertainty. For the discrete period case (meaning there is a finite list of periods under investigation), we show that the worst-case fixed period power provides a lower bound on the power of an unknown-period hypothesis test after Bonferroni correction (183,Eq. (S105)).

The free period model is most relevant to our results on continuous period uncertainty (Sec. 3.3). We show numerically that there is correlation between the power of the fixed and free period models (Fig. 3B). When designs optimized for fixed period power were tested using free period power, they continued to show improvements relative to equispaced (Fig. 3A). Since the free period power was not analytically tractable, we derived a lower bound on a relaxation of the free-period power (Supp. Sec. S2). Designs that were constructed using the free-period power bound as an objective performed similarly to the existing fixed-period designs (Fig. 3A). Based on our new analysis, our revised manuscript recommends that experiments with continuous period uncertainty use worst-case fixed-period as a heuristic for design construction and then evaluating performance using the free-period power (line 232).

Minor points

2. Top of page 3: Citations 20,21 are for two text books - can the authors be more specific about what is meant? Is there a specific page rage? Are these for definition of "optimal performance" or do they provide a proof of the claim that equidistant spacing is optimal for fixed-period cosinor analyses? It is unclear to the reader.

We are referring to the proof that equispaced designs are D-optimal for trigonometric regression. We have updated Ref. 20 to include a chapter reference and moved Ref 21 to only appear in the supplemental material where it is referenced for a specific theorem on E-optimality.

3. Is the direction of inequality in equation (16) correct? I would have expected the chosen lambda to out perform the one in equations 12-14 instead of underperform. Why not just use equation 12 instead then?

This was a shortcoming of our notation. The inequality in Eq. (16) gives an upper bound on the smallest eigenvalue of B^{-1}, the objective function we seek to maximize. We have updated the notation to separately define the objective function in Eq. (15) and then state the upper bound on the optimal solution to a maximization problem in Eq. (16).

4. Section 5.2 Brute-force: It's not obvious to my why we can represent these as binary vectors - i.e., why is it never the case that two or more samples could be taken at the same time? Clearly in some applications, that wouldn't be done for practical reasons, but for others, it's quite common to take independent samples at the same time point

This confusion likely arises because of an assumption that was left implicit in our initial submission. We assume throughout our work that samples are collected from a single individual or biological specimen (e.g. a blood collection). For such cases, samples taken at the same time point would not be independent and would not be valid for use in cosinor analysis. In cases where independent sampling at the same time point is possible, this assumption could be removed, and the search would run over k-ary rather than binary necklaces. We agree that this simplifying assumption was not clear from our original presentation and have made the assumption of equal sample sizes between measurements explicit on lines 363 and 388.

5. Lemma S1.4: V_ℓ is not a subspace (it does not contain 0, for example). It looks like you can just redefine V_ℓ to be the subspace of V_u that is orthogonal to V_r. For the second equality, it's worth noting that the $Q(Q^TQ)^{-1}Q^T$ is easily verified to be an orthogonal projection matrix ($P^2 = P$ and $P = P^T$) from the definition of Q. Without that, computing the null space is not sufficient.

We thank the reviewer for pointing out this oversight. We have made the suggested correction to the definition of V_ℓ and verified that the conclusion of the proof still holds.

6. Lemma S1.7: Why are P_A and P_B commuting? It appears to need another assumption.

The assumption of commuting projectors was missing from our original proof, we have stated this assumption explicitly in our revised manuscript. We have also confirmed that the additional assumption is satisfied when we apply Lemma S1.7 in the proof of Lemma S1.8. In particular, the commutation is trivial since $(I-P_u)(P_u-P_r) = 0 = (P_u-P_r)(I-P_u)$.

7. maximziation $\rightarrow$ maximization (section S1.2)

We have corrected the typo.

8. Figure S5: No (C) in figure caption? Hard to decipher these plots: I take it that the pairs on the right hand side ("1,2") are the frequency priors and the fmin=1 and fmax = the second number of this? Also, please spell out for the reader what the Nyquist frequency is here (and elsewhere in the paper as applicable). N = 12, so the Nyquist frequency is 6 and the integer multiple 12 is also affected? Worth saying for the reader as I, at least, always have to think through periods versus frequencies, and the sample size (and hence Nyquist frequency) is not constant throughout this paper. Why don't all these subfigures (of B and C) have the same numbers of points drawn? Is there some filtering for a significant p-value or something? If so, I find that questionable when considering the biasedness of amplitude estimates, in particular. Moreover, amplitude estimates appear biased upwards, especially at low amplitude. I guess that this is inherent to the cosinor model and amplitude being positive, but with the discussion of bias, this needs a comment and probably a clarification that bias is being used to refer to acrophase estimates only.

We have added a definition of the Nyquist rate to the background section (line 72) and remind the reader of its definition in relevant sections (lines 221, 238, 273, 105). We have updated the title of the figure (Figure S6 in the revised manuscript) to clarify that we are referring to bias after filtering for statistical significance and clarified that panels (B-C) share a caption.

9. Monte Carlo is dismissed as infeasible for these study designs, presumably due to computational limits. Perhaps it would be possible to compare computational costs to PowerCHORD?

We have added a figure that compares computational costs for Monte Carlo power analysis, exact F-test power, and the free-period power bound (Supp. Fig. 8).

Reviewer 2

Major points

1. My major concern is with the use of the cosinor model, which assumes symmetric, stationary and sinusoidal waveforms. While this model is indeed broadly used in the literature, typical usage has been limited to detecting rhythms and periodicity of the rhythms. However, I'm not sure why a cosinor model is appropriate for the more nuanced question that the authors are asking here, i.e., how to space out measurements of the rhythms over time in order to optimize power. It is well known that biological rhythms tend to be non-stationary, with amplitude, time-period and phase velocity varying with time. This has been demonstrated carefully, particularly for the circadian clock which is referred to multiple times in this study. Older studies have demonstrated non-stationarity in activity and body temperature rhythms (PMID: 15038991). Recent studies show this even at the molecular level, using careful gene expression measurements (mouse tissues in Tong et al https://doi.org/10.1101/2025.04.03.647044 and mouse cell-lines in Nikhat et al; https://doi.org/10.1101/2024.06.30.601408). For instance, Figure 2F in Tong et al clearly show that phase velocity in not constant across time. Figure 4D in Nikhat et al shows how the phases at 8 and 32 hours post synchronization are quite different, though one might expect them to be identical under a 24-hour cosinor model. Therefore, the authors should carefully discuss why a cosinor model might still be suitable in the light of these experimental datasets.

We appreciate you bringing this point to our attention and recognize that many readers may have similar concerns about our use of the cosinor model. Our revised manuscript includes justification for our use of the cosinor model (line 103) which we summarize briefly below.

The focus of our work is rhythm detection under the assumption that the study has limited prior knowledge and cannot predict how the system might deviate from the cosinor model. Based on this assumption, we optimize for the simplest case and then confirm that rhythms remain detectable in more complex situations where the system departs from model assumptions. In our revised manuscript, we verify by simulation that the designs continue to perform well when applied to several types of more complex signals (Supp. Fig. 4). Explicit optimization for non-cosinor signals is important, but it would likely require data from an initial study which would then be used for iterative/Bayesian study design. Similar methods to the ones we present could be useful for this, but we view this as an extension of our work.

2. In Theorem 3.2, the authors prove that if $N > 3$ equispaced measurements can be made per cycle of a rhythm with known frequency, this design optimizes worst-case power. How strongly does this result depend on the underlying assumptions of the cosinor model (as stated above)? For instance, if the oscillation had a non-varying,

fixed 24 hour time-period across cycles, but the phase velocity varied as a function of time, would this result still hold? Intuitively it would seem one would need more closely spaced measurements where phase velocity is faster, and less measurements where velocity is slower.

Our new simulations (Supp. Fig. 4) address the robustness of equispaced designs to non-sinusoidal rhythms. For most of the signal families, equispaced designs outperformed all 100 randomly generated designs, and for the signal with least resemblance to the cosinor model, their performance was comparable to the random designs. Since all of the simulations generated acrophases from a uniform prior, we conjectured that equispaced optimality could be a consequence of the phase invariant prior, and not strictly related to the detailed assumptions of the cosinor model. We prove a quantitative version of this statement in Sec. S1.6, confirming our intuition that equispaced optimality holds in greater generality than what was demonstrated in our initial submission.

Your intuition about phase-dependent velocity is likely correct and could be applicable to studies that perform iterative experimental design (i.e. choosing when to collect measurements based on the data that has already been collected in an earlier stage of the study). Such approaches are common in Bayesian optimal experimental design, and would be a valuable extension of our work.

3. There has been significant effort in recent times to go beyond the cosinor-type models and model non-stationary rhythms with irregularly sampled data. Some examples are wavelet transforms(PMIDs: 28569118 and 35610418) and Gaussian Processes (PMID: 37769241) that have been used on circadian rhythm analysis. Indeed, the second article carefully demonstrated that accounting for non-stationarity significantly improves waveform and rhythm detection over methods like cosinor and Lomb-Scargle Periodograms. Therefore, would it be possible to extend the power calculations in this work to these non-stationary models – at least numerically, if not with closed form expressions? To me it seems fundamentally important to account for non-stationarities in the underlying data when the question of measurement spacing is of primary interest.

Power optimization is considerably more challenging for models where closed-form expressions for the null and alternative distributions are unavailable. At the same time, we recognize that more complex statistical models necessitate the consideration of such scenarios. To this end, we have extended our approach to power optimization to include permutation-based rhythm detection (Sec. 3.3.1; Supp. Sec. S2).

Our new results on permutation tests have two implications for our study. First, they legitimize our heuristic approach to continuous period uncertainty (Sec. 3.3). Design generated by either a rigorous permutation method or the simpler heuristic from our initial submission both improve free-period power relative to equispaced near the Nyquist rate (Fig. 3).

FACULTY OF ARTS & SCIENCE
Bahen Centre, 40 St. George St., Room 6290, Toronto, ON M5S 2E4 Canada
Tel: +1 416 978-3323 • Fax: +1 416 978-4107 • www.math.utoronto.ca

Second, permutation analysis provides a framework for extending power analysis to more complex statistical models. Any rhythm detection method that relies on a quadratic test statistic could apply our permutation-based approach (Supp. Sec. S2) to optimize power.

4. How strongly are the results of this work dependent on the assumption of homoscedastic noise in Eq. (1)? In reality, as can be observed in all the references above, noise at different time points in biological rhythms does not have equal variance. This is not just a technical issue, but for circadian clocks, also arises from the underlying biology of transcription (Nikhat et al; https://doi.org/10.1101/2024.06.30.601408). Could the authors incorporate heteroscedasticity, at least in their simulated datasets and check how well their main results hold?

We have investigated the robustness of equispaced designs to heteroscedastic noise through numerical simulations. Our results show that equispaced designs continue to perform well even when the noise variance varies periodically, as long as the acrophase prior remains uniform. To better understand this observation, we have extended the proof of our main optimality result to a broader class of convex, phase-invariant objective functions (Supp. Sec. S1.6). This analysis highlights that the lack of prior knowledge about the acrophase at the study design stage is one of the primary reasons for the optimality of equispaced designs.

5. Related to the previous question, I could not understand how the authors estimated sigma, the s.d. of the Gaussian white noise. This term seems important even in the expression for power in Equations 9 and 10, hence a clearer explanation of how it is estimated would be very helpful. This is especially so because at the time of a power calculation, before the actual data is collected, one would not know how much noise will exist in the data.

This is an important point and we have elaborated on our treatment of the signal-noise ratio in our revised manuscript (line 126). In general, the noise strength does not need to be estimated in order to optimize the power because it only rescales the objective function and therefore does not affect the location of the critical points. Noise strength would become relevant in iterative study designs, where signals of lower amplitude may require higher priority (for example, by measuring more frequently at phases with lower average amplitude). As these considerations lie outside the scope of our present work, we have not pursued them further here.

6. Finally, a major limitation of the current manuscript is a lack of application to any experimental dataset. As a result, I found it hard to understand how exactly an experimentalist can use PowerCHORD to set up a new experiment – what would be the input data and data format required to run PowerCHORD, parameters, what

needs to be prior knowledge etc. For this algorithm to be practically useful, I would strongly urge the authors to provide a worked out example using an experimental dataset. Adding a flowchart in the main text explaining the main steps to be followed to implement the code would also help improving the readability of the manuscript and ease of practical use. While the code is available on Github, this is also not very easily accessible to the potential user, and neither is the "examples/power_analysis.Rmd" file very easy to understand.

We thank the reviewer for this comment. Upon reflection, we realized that our original title could have suggested that the manuscript's primary focus was on software implementation (many package/software articles follow the standard "Acronym: a method for ..."). We feel that our work is more centered on understanding the optimization problem itself. In instances where numerical methods are required, our optimization methods require minimal user input to specify study parameters. Effectively, we are working in a context where minimal apriori knowledge is assumed, so there is very little information to be gained from having input from a specific study. To prevent any misunderstanding, we have updated the title to more clearly reflect the focus on the optimization problem rather than on the software itself. To further emphasize the focus of our work, we've made similar changes to the tone of our writing throughout the manuscript and emphasizes the scope of our work in the introduction (line 36).

We have revised our GitHub examples to provide clearer guidance on how to analyze and optimize the power of an initial study and have specified the inputs for each algorithm throughout the manuscript (lines 154 190 219 353)

Minor points

1. I found the choice of alphabets in the equations a bit confusing. The lambda in Eq. (9) refers to the non-centrality parameter, but does the lambda in Eq. (12) represent the eigenvalues of the matrix B^{-1}?

We agree that distinction is important to emphasize, since the noncentrality parameter and minimal eigenvalue of B^{-1} are monotone functions of one another but not identical. Since lowercase λ is standard notation for noncentrality parameter, we have updated our notation to use ξ for matrix eigenvalues to avoid any confusion.

2. The manuscript in its current form will not be easily accessible to most chronobiology researchers, even those with a more computational background. Given that the results are quite interesting and of potential use to a large community of researchers, I would suggest adding some less technical but more conceptual notes in the text, perhaps as

Explanatory Boxes. For instance, some intuitive explanations of the three optimization methods used would significantly enhance readability. Similarly, some intuitive explanations of why irregular designs are better than equispaced designs would be very helpful. Currently the manuscript provides no conceptual intuition and the interesting results are somewhat lost behind the mathematical formalism.

We have completely restructured our results section in an effort to make our work more accessible to experimentalists. In particular, theoretical results are presented based on the type of experiment to which they are relevant instead of being presented all at once in a separate section. Results with the concrete experimental applications now appear earliest in the results section. To further improve the accessibility of our work, we have extended the background section of our manuscript and revised the tutorial file.

FACULTY OF ARTS & SCIENCE
Bahen Centre, 40 St. George St., Room 6290, Toronto, ON M5S 2E4 Canada
Tel: +1 416 978-3323 • Fax: +1 416 978-4107 • www.math.utoronto.ca
