# Supplementary material for: Optimization of experimental designs for biological rhythm discovery
Source: PLoS Comput Biol. 2025 Nov 10;21(11):e1013662. doi: 10.1371/journal.pcbi.1013662 (PMC12617917; doi:10.1371/journal.pcbi.1013662)
Supplement: Attachment — Submitted filename: response_auresp_2.pdf [file pcbi.1013662.s012.pdf]

Mathematics
UNIVERSITY OF TORONTO

Adam Stinchcombe
Department of Mathematics, University of Toronto
40 St. George Street, Rm 6290
Toronto, ON M5S 2E4, Canada
stinch@math.toronto.edu
(416) 946-7193

RE: manuscript PCOMPBIOL-D-25-00228 October 20, 2025

Dear Dr. Robinson-Rechavi,

We are delighted to hear that our revised manuscript was received positively by both reviewers. We have made minor revisions to address the remaining points, as detailed below.

Cheers,

Adam Stinchcombe

Adam Stinchcombe
Associate Professor of Mathematics
on behalf of all co-authors

Reviewer 1

1. My only new comment is that many figures come across as low-resolution and with significant compression artefacts in the version I am reviewing and I would advise the authors to make sure higher quality figures are available at publication.

We have regenerated all figures as vector graphics to ensure they are legible in both PDF and HTML versions of our manuscript.

Reviewer 2

1. The justification for use of a cosine model in Line 103 of the updated manuscript is fair, but not very informative. While Supp Fig 4 explores non-sinusoidal waveforms, this is not really the core issue in real datasets. Rather, the pervasive non-stationarity of the datasets is more important, for example, large variations in consecutive peak to peak distances in a time series dataset. While I understand that analysing non-stationary rhythms may not be within the scope of this manuscript, the authors should at least provide a more detailed discussion of this point such that readers are informed about the various assumptions and limitations involved with cosinor models, which are inherently stationary.

We agree that it's important to consider period non-stationarity in biological rhythms. Since this is beyond the scope of our work, we have placed more emphasis on ref 26 by citing it separately from other alternatives to the cosinor model (line 128 of the revised manuscript). We hope that the additional emphasis on this topic will encourage readers to think critically about the robustness of their experimental designs to realistic non-cosinor signals.

2. Related to the point above, the authors respond to the question of non-stationarity by saying that "Power optimization is considerably more challenging for models where closed-form expressions for the null and alternative distributions are unavailable." This seems to suggest that current methods for analysing non-stationary rhythmic datasets do not provide closed form expressions for the null and alternative distributions. This is however not true – Gaussian Processes provide closed form solutions for both distributions, since both are multi-variate normals but with appropriate covariance matrices (see for example PMID 37769241; ref 24 in the author's updated manuscript). The authors might want to comment on this point for clarity.

Our use of the terminology "closed-form expressions for the null and alternative distributions" in our earlier comment was ambiguous. To clarify, we were referring specifically

to the distribution of the test statistic under the null and alternative models. In the case of Gaussian Processes in ref 24, the marginal log likelihood has a known distribution for any given kernel under the null and alternative models as you pointed out (Eq. 18 of PMID 37769241 Supplementary material). Since the Bayes factor on the other hand is computed by optimizing the kernel under the null and alternative models, it can be understood as a ratio of extreme values of correlated random variables and therefore has no closed form distribution. Since the Bayes factor is used as the test statistic, power optimization would likely require an approximate or fully numerical approach.

FACULTY OF ARTS & SCIENCE
Bahen Centre, 40 St. George St., Room 6290, Toronto, ON M5S 2E4 Canada
Tel: +1 416 978-3323 • Fax: +1 416 978-4107 • www.math.utoronto.ca
